# Coordinated power management strategy for reliable hybridization of multi-source systems using hybrid MPPT algorithms

**DOI:** 10.1038/s41598-024-60116-4

**Published:** 2024-05-04

**Authors:** Djamila Rekioua, Zahra Mokrani, Khoudir Kakouche, Adel Oubelaid, Toufik Rekioua, Mohannad Alhazmi, Enas Ali, Mohit Bajaj, Shir Ahmad Dost Mohammadi, Sherif S. M. Ghoneim

**Affiliations:** 1 Laboratoire de Technologie Industrielle et de l’Information, Faculté de Technologie, Université de Bejaia, 06000 Bejaïa, Algeria; 2 Electrical Engineering Department, College of Applied Engineering, King Saud University, P.O. Box 2454, 11451 Riyadh, Saudi Arabia; 3 Centre of Research Impact and Outcome, Chitkara University Institute of Engineering and Technology, Chitkara University, Rajpura, Punjab 140401 India; 4 Division of Research and Development, Lovely Professional University, Phagwara, Punjab India; 5 Department of Electrical Engineering, Graphic Era (Deemed to be University), Dehradun, 248002 India; 6 Hourani Center for Applied Scientific Research, Al-Ahliyya Amman University, Amman, Jordan; 7 Graphic Era Hill University, Dehradun, 248002 India; 8 Department of Electrical and Electronics, Faculty of Engineering, Alberoni University, Kohistan, Kapisa Afghanistan; 9 Department of Electrical Engineering, College of Engineering, Taif University, 21944 Taif, Saudi Arabia

**Keywords:** Photovoltaic, Wind turbine, Hybrid MPPT, Power management control, Hybrid energy storage, Optimization, Energy science and technology, Engineering, Mathematics and computing

## Abstract

This research discusses the solar and wind sourcesintegration in aremote location using hybrid power optimization approaches and a multi energy storage system with batteries and supercapacitors. The controllers in PV and wind turbine systems are used to efficiently operate maximum power point tracking (MPPT) algorithms, optimizing the overall system performance while minimizing stress on energy storage components. More specifically, on PV generator, the provided method integrating the Perturb & Observe (P&O) and Fuzzy Logic Control (FLC) methods. Meanwhile, for the wind turbine, the proposed approach combines the P&O and FLC methods. These hybrid MPPT strategies for photovoltaic (PV) and wind turbine aim to optimize its operation, taking advantage of the complementary features of the two methods. While the primary aim of these hybrid MPPT strategies is to optimize both PV and wind turbine, therefore minimizing stress on the storage system, they also aim to efficiently supply electricity to the load. For storage, in this isolated renewable energy system, batteries play a crucial role due to several specific benefits and reasons. Unfortunately, their energy density is still relatively lower compared to some other forms of energy storage. Moreover, they have a limited number of charge–discharge cycles before their capacity degrades significantly. Supercapacitors (SCs) provide significant advantages in certain applications, particularly those that need significant power density, quick charging and discharging, and long cycle life. However, their limitations, such as lower energy density and specific voltage requirements, make them most effective when combined with other storage technologies, as batteries. Furthermore, their advantages are enhanced, result a more dependable and cost-effective hybrid energy storage system (HESS). The paper introduces a novel algorithm for power management designed for an efficient control. Moreover, it focuses on managing storage systems to keep their state of charge (SOC) within defined range. The algorithm is simple and effective. Furthermore, it ensures the longevity of batteries and SCs while maximizing their performance. The results reveal that the suggested method successfully keeps the limits batteries and SCs state of charge (SOC). To show the significance of system design choices and the impact on the battery’s SOC, which is crucial for the longevity and overall performance of the energy storage components, a comparison in of two systems have been made. A classical system with one storage (PV/wind turbine/batteries) and the proposed system with HESS (PV/wind turbine system with batteries). The results show that the suggested scenario investigated with both wind and solar resources appears to be the optimum solution for areas where the two resources are both significant and complementary. The balance between the two resources seems to contribute to less stress on storage components, potentially leading to a longer lifespan. An economical study has been made, using the Homer Pro software, to show the feasibility of the proposed system in the studied area.

## Introduction

Renewable energy technologies are rapidly being implemented in rural regions^1–3^. Nonetheless, because to the variable nature of renewablesources, MPPT algorithms are essential to maximize power output. Various MPPT methods are applied to obtain the maximum power point of solar panels^4–18^ and wind turbines^19–29^. Despite the fact that they all aim to obtain more power, they all operate in distinct methods. In the literature, a classification has been developed to clarify the various techniques, which include classical, advanced and hybrid ones. Classical approaches can be classified as direct or indirect. Advanced approaches are divided into two categories: artificial intelligence and bio-inspired methodologies. Hybrid MPPT (HMPPT) has been widely employed in recent years. It can be a mixture of two traditional MPPT methods^30^, a classical with an advanced approach^8,31^, or two advanced methods^32^. In PV systems, multiple approaches can be used. The P&O approach is widely utilized because of its ease of use. However, it has the disadvantage of oscillations, which cannot be totally removed^12,15^. For advanced methods, the FLC, artificial neural networks (ANN) and sliding mode control (SMC) are the most used^26–28^. Also, in wind turbines, the P&O algorithm^33^ is the most frequently employed, along with other techniques such as Optimal Torque Control (OTC)^32^, Tip Speed Ratio (TSR)^33^, Power Signal Feedback (PSF), the FLC, and particle swarm optimization (PSO)^28,34^.

Energy storage systems (ESSs) are crucial for maintaining optimal power balance in hybrid PV/Wind turbine systems. The selection of storage technology is influenced by system requirements, budget constraints, and a rigorous examination of benefits and drawbacks^35–41^. There are various technologies for ESSs.Batteries are extensively utilized becauseof their low cost and ease of installation^36–38^. Also, supercapacitors offer advantages like as quick charging and discharging but come with constraints like low energy density, high cost, and limited lifespan^39–41^. Power management control (PMC) is important for the successful and efficient operation of multiple energy storage devices in a hybrid renewable system with multi-storage. Several research publications have been published on the power management of hybrid PV/wind turbine systems utilizing storage or multi-storage technology^42–50^.Other important works emphasize the importance of effective power management strategies in hybrid PV/wind systems utilizing various storage technologies, highlighting the significance of optimizing energy flow, enhancing system stability, and improving overall efficiency and reliability^51–63^.We can’t mention all the articles, because there are so many, but some are very important to mention. These are in general reviews on Control, Energy Management Approaches in Micro-Grid Systems or hybrid renewable systems^64–70^. These reviews offer valuable insights into various control strategies, energy management approaches, and optimization algorithms in micro-grid and hybrid renewable energy systems. They provide a state-of-the-art research in this field and highlight key challenges and opportunities for future development^71,72^. Some details are given in “Related works” section.

In this paper, a Power Management Control (PMC) system to control the different sources and the various storage systems is provided. The use of this PMC has been applied in an area with considerable potential of solar irradiation and wind speeds, for different profiles. Weather conditions and geographic consideration have been taken account and due to the proposed PMC, high system performance is obtained throughout the year. When comparing the proposed system PV/ wind turbine with hybrid storage (batteries/SCs) to existing systems with one storage (PV/Wind turbine/batteries), the batteries were less stressed, which increases the performance of the system thus a great advantage that brings the proposed system. The findings by simulation show the efficacy of the proposed PMC. The work’s purpose is to show the feasibility of solar and wind energy systems optimized by a hybrid power maximizing method and incorporate several storage systems and a power management system. In our work, we have applied the proposed power management strategy in a hybrid renewable energy system combining solar photovoltaic (PV) and wind power sources and applied it in the area of Bejaia (Algeria), which has a great potential of solar irradiance and wind speeds. These variable weather conditions highlight how the strategy adapts to dynamic input sources. The application of the proposed system can be tooff-grid power systems (our case), to electric vehicle charging stations, remote communication stations, smart microgrid integration.

An economical study has been made, using the Homer Pro software, to show the feasibility of the proposed system in the studied area.

This study marks a significant stride towards sustainability, efficiency, and energy autonomy for customers.

## Related works

A summary of significant research related PMC in PV and wind systems with storage and hybrid storage, is presented in Table 1 below. These studies primarily focus on control strategies, energy management approaches, and optimization techniques in micro-grid and hybrid PV/wind systems incorporating battery storage or hybrid energy storage.

**Table 1:** Some important works related on PMC with storage.

Study	Year	Description
^42^	2011	Authors demonstrate the advantages of thermal energy storage in hybrid systems for reducing the size of battery bank. But there is no comparison of the proposed systems with alternative approaches or technologies and there no optimization study
^43^	2012	Authors give an analysis of power management strategies for hybrid PV/wind systems, tacking account various energy storage technologies and control methods
^44^	2015	The paper focuses on developing a supervisor control system to supply an electric vehicle. The battery bank serves as an energy storage mechanism, storing excess energy generated by the PV andProton Exchange Membrane Fuel Cell(PEMFC) systems for later use when demand exceeds supply
^45^	2017	Authors present a comparative study of different power management strategies for hybrid PV/wind systems with battery storage, analyzing their impact on system efficiency
^46^	2017	It is proposed a decentralized PMC for hybrid PV/wind systems with distributed energy storage, aiming to improve system robustness and efficiency
^47^	2020	Authors introduce a PV system with battery and supercapacitor. Hybrid MPPT has not been taken account and the application is given only to PV systems
^48^	2020	The study identifies the necessity for hybrid power generation from solar PV and wind. They take account only on batteries for storage and conclude on the importance of optimizing the battery storage to reduce overall system cost
^49^	2020	Authors propose the integration of multiple energy storage devices into hybrid energy storage systems within standalone micro grids. AFLC algorithm is introduced for standalone DC microgrids with multiple energy storage
^50^	2020	Proposed a PMC for PV/wind system with battery storage, focusing on optimizing energy flow and enhancing system stability
^51^	2020	Investigated the feasibility of utilizing flywheel energy storage in hybrid PV/wind systems and proposed a corresponding power management strategy for optimal operation
^52^	2020	Explored the optimization of hybrid PV/wind systems with multi-storage technology using evolutionary algorithms and proposed an adaptive power management framework
^53^	2020	Investigated the impact of different power management approaches on the stability and reliability of hybrid PV/wind systems with integrated energy storage systems
^54^	2021	The paper proposes a techno-economic design of an off-grid solar/wind system with a hybrid energy storage system. The proposed approach is validated through simulations using MATLAB/Simulink
^55^	2022	Investigated the impact of different power management strategies on the performance of PV/wind systems with multi-storage technology
^56^	2022	Developed a predictive power management algorithm for hybrid PV/wind systems with thermal energy storage, focusing on improving energy utilization and grid integration
^57^	2023	Authors introduce a BMS specifically designed for a modified interlinking converter within a hybrid AC/DC microgrid. The results demonstrate appropriate performance in both grid-connected and standalone modes, but the optimization has not been taken account
^58^	2023	The paper addresses the critical issue of PMC in autonomous hybrid systems, particularly focusing on challenges associated with optimizing energy sources and backup systems, especially under heavy loads or low renewable energy output conditions
^59^	2023	Authors integrate solar and wind energy with a PMC and multi storage, with a mono MPPT and there is no cost–benefit analysis to assess the economic viability of the proposed energy management scheme
^60^	2023	The paper proposesa new multi-stage PMC. A fuzzy PMC is employed to manage the power flow electric
^61^	2023	Developed a novel power management algorithm for hybrid PV/wind systems integrated with both battery and supercapacitor storage, emphasizing energy optimization
^62^	2023	Explored the integration of pumped hydro storage in hybrid PV/wind systems and proposed an adaptive power management approach to enhance system performance
^63^	2023	Investigated the use of compressed air energy storage in hybrid PV/wind systems and proposed an intelligent power management strategy to maximize system benefits

## Proposed hybrid PV/wind turbine with hybrid energy storage system

**Figure 1:**
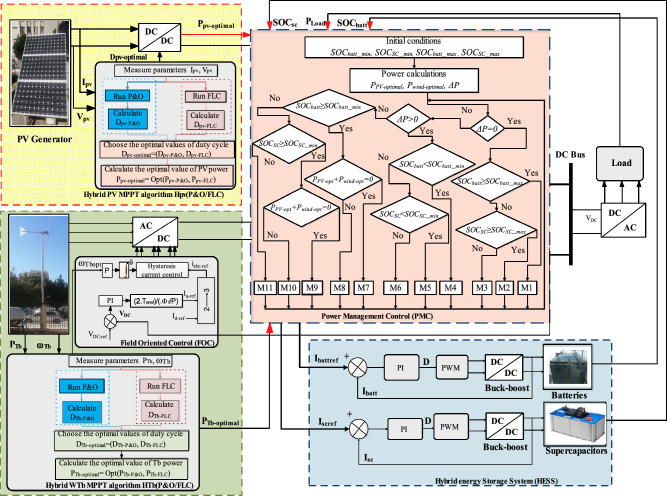
Proposed Optimized PV/Wind system with hybrid energy storage system.

The studied system consists of four distinct parts (Fig. 1). First, there is a PV generator with a DC/DC converter aimed at maximizing output power. This is achieved using a hybrid MPPT algorithm HPV (P&O/FLC), combining P&O and FLC methods. The second block features a wind turbine and a permanent magnet synchronous generator.

To maximize wind power, the proposed approach is HTb (P&O/FLC), combining P&O and FLC methods. The third block consists of a hybrid (batteries/SCs) storage system. Battery technology enables long-term energy storage, however, supercapacitors are capable of absorbing current changes, lowering the risk to batteries. Finally, the fourth block is the PMC system, where the inputs are optimized PV (P_pv-optimal_) and wind powers (P_Tb-optimal_), the SOC of batteries (SOC_Batt_) and SCs (SOC_SC_) and the load power (P_Load_).

## Measurementof solar radiation and wind speeds

**Figure 2:**
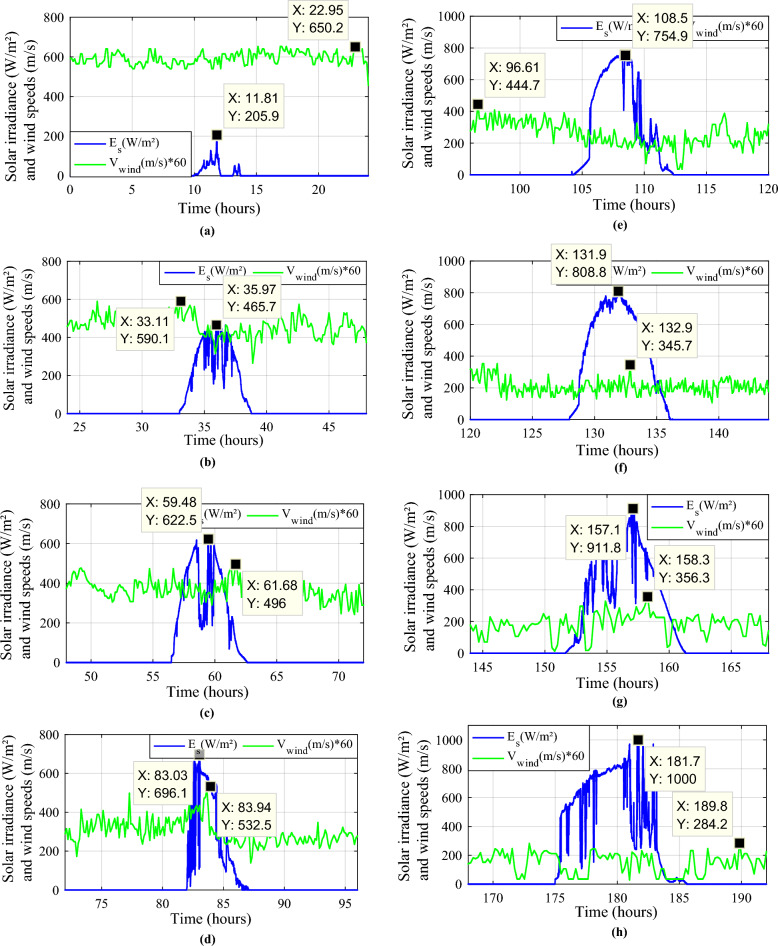

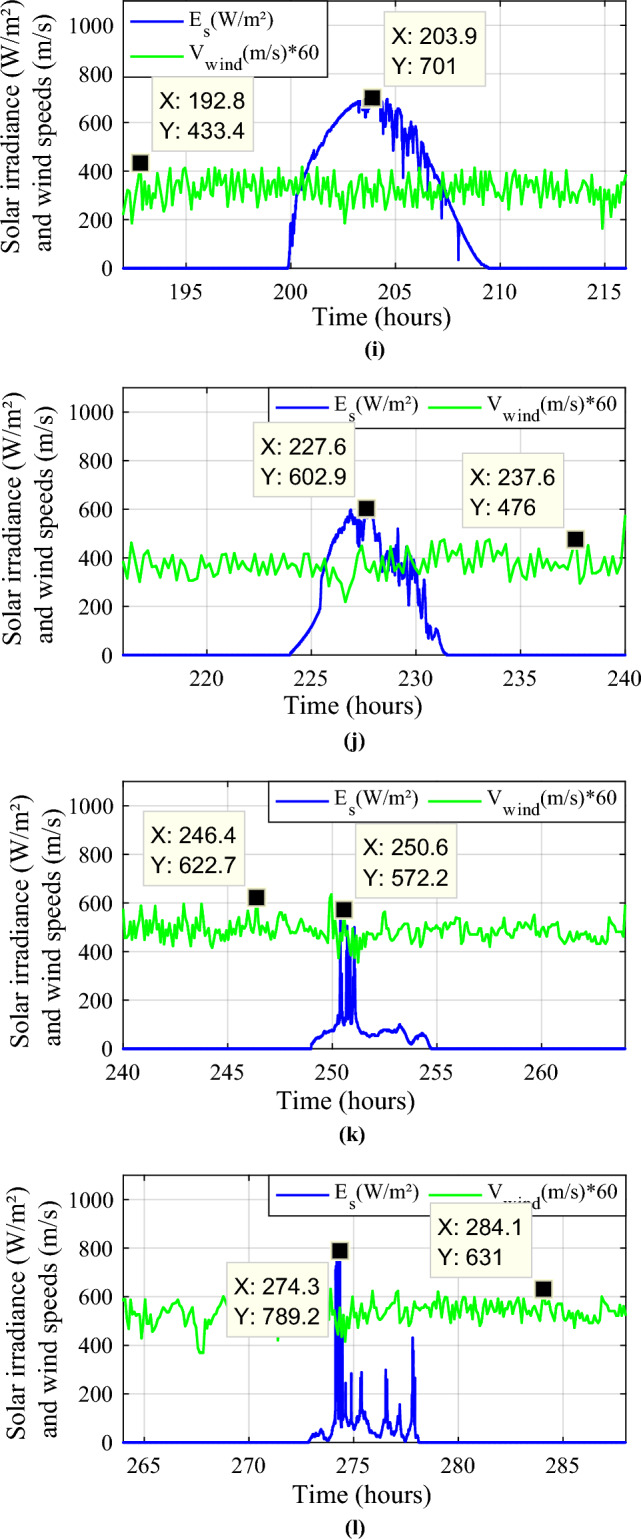
Irradiance and wind speed measurements. (**a**) Profile 1. (**b**) Profile 2. (**c**) Profile 3. (**d**) Profile 4. (**e**) Profile 5. (**f**) Profile 6. (**g**) Profile 7. (**h**) Profile 8. (**i**) Profile 9. (**j**) Profile 10. (**k**) Profile 11. (**l**) Profile 12.

Measurement acquisition equipment was used to measure the solar irradiation, temperature and wind speeds where the solar irradiance and wind speeds are complementary all the year. It is essentially composed of sensors in order to transfer the different signals to a data processing interface and then to a PC where they will be displayed using ACQUIsol software in real-time. The measurements have been made in the studied site, the different measured profiles for each month of a year have been simulated (Fig. 2).

To test the effectiveness of the proposed energy management strategy, extensive numerical simulations were carried out under MATLAB/Simulink environment. Runge Kutta of 4th order is used as a solver with a step of 1e−5.

The Table 2 below summarizes the used simulation details.

**Table 2:** Parameters simulation details.

Parameters	Value
D	96 days
Ts	1e−4
Solver	RK-ode4
Solver type	Fixed

## System component modeling

The different components are a PV generator with a DC/DC converter, a wind turbine, a permanent magnet synchronous generator (PMSG) and a hybrid (batteries/SCs) storage system^72–74^. Each component has been modeled before its simulation (Table 3).

**Table 3:** Parametrized mathematical models of system components.

Components	Diagram or equivalent circuit	Equations	Refs	Parameters	
PV generator		${\text{I}}_{{{\text{pv}}}} = {\text{ n}}_{{\text{p}}} \cdot {\text{I}}_{{{\text{ph}}}} - {\text{n}}_{{\text{s}}} \cdot {\text{I}}_{{{\text{sat}}}} \left( {{\text{e}}^{{\frac{{{\text{q}}\left( {{\text{V}}_{{{\text{pv}}}} + {\text{R}}_{{\text{s}}} \cdot {\text{I}}_{{{\text{pv}}}} } \right)}}{{{\text{n}}_{{\text{s}}} \cdot {\text{A}} \cdot {\text{K}} \cdot {\text{T}}_{{\text{j}}} }}}} - 1} \right) - {\text{I}}_{{{\text{sh}}}}$	^32,58,61^	P_PV_	80 W_p_
				I_mpp_	4.58 A
				V_mpp_	17.5 V
				I_sc_	4.95A
				V_oc_	21.9 V
				α_sc_	3.00 mA/°C
				β_oc_	− 150.00 V/°C
Wind turbine		$$\left\{ {\begin{array}{*{20}l} {{\text{P}}_{{{\text{Tb}}}} = \left( {1/2} \right) \cdot {\text{C}}_{{\text{p}}} \cdot {\uprho } \cdot {\uppi } \cdot {\text{R}}_{{{\text{Tb}}}}^{2} \cdot {\text{V}}_{{{\text{wind}}}}^{3} } \hfill \\ {{\text{P}}_{{{\text{Tb}} - {\text{opt}}}} = \left( {1/2} \right) \cdot {\text{C}}_{{{\text{pmax}}}} \left( {{\uplambda }_{{{\text{opt}}}} } \right) \cdot {\uprho } \cdot {\uppi } \cdot {\text{R}}_{{{\text{Tb}}}}^{2} \cdot {\text{V}}_{{{\text{wind}}}}^{3} } \hfill \\ \end{array} } \right.$$ $$\left\{ {\begin{array}{*{20}c} {{\text{T}}_{{{\text{Tb}}}} = \left( {1/2} \right).{\text{C}}_{{\text{p}}} .{\uprho }.{\uppi }.{\text{R}}_{{{\text{Tb}}}}^{5} .\frac{{{\upomega }_{{{\text{Tb}}}}^{2} }}{{{\uplambda }_{{}}^{3} }}} \\ {{\text{T}}_{{{\text{Tb}} - {\text{opt}}}} = \left( {1/2} \right).{\text{C}}_{{{\text{p}} - {\text{opt}}}} .{\uprho }.{\uppi }.{\text{R}}_{{{\text{Tb}}}}^{5} .\frac{{{\upomega }_{{{\text{Tb}}}}^{2} }}{{{\uplambda }_{{{\text{opt}}}}^{3} }}} \\ \end{array} } \right.$$ ${\text{J}} \cdot \left( {{\text{d}}\upomega _{{{\text{Tb}}}} /{\text{dt}}} \right) = {\text{T}}_{{{\text{Tb}}}} - {\text{T}}_{{{\text{em}}}} - {\text{f}} \cdot\upomega _{{{\text{Tb}}}}$	^33,58^	Blades	03
				λ_opt_	8.1
				C_p_	0.48
				V_w-Rated_	12.5 m/s
				V_w-cut-in_	3.4 m/s
				R_Tb_	1.05 m
PMSG		$$\left\{ {\begin{array}{*{20}c} {{\text{V}}_{{{\text{sd}}}} = {\text{R}}_{{{\text{st}}}} {\text{I}}_{{{\text{sd}}}} + {\text{L}}_{{\text{d}}} \left( {\frac{{{\text{dI}}_{{{\text{sd}}}} }}{{{\text{dt}}}}} \right) - {\text{L}}_{{\text{q}}} {\omega I}_{{{\text{sq}}}} } \\ {{\text{V}}_{{{\text{sq}}}} = {\text{R}}_{{{\text{st}}}} {\text{I}}_{{{\text{sq}}}} + {\text{L}}_{{\text{q}}} \left( {{\text{dI}}_{{{\text{sq}}}} /{\text{dt}}} \right) + {\text{L}}_{{\text{d}}} {\omega I}_{{{\text{sd}}}} + {\Phi }_{{\text{f}}} {\upomega }} \\ {{\upomega } = {\text{P}}\Omega } \\ \end{array} } \right.$$ ${\text{T}}_{{{\text{em}}}} = \left( {3/2} \right)\left[ {{\Phi }_{{\text{f}}} .{\text{I}}_{{{\text{sq}}}} + \left( {{\text{L}}_{{\text{d}}} - {\text{L}}_{{\text{q}}} } \right).{\text{I}}_{{{\text{sd}}}} .{\text{I}}_{{{\text{sq}}}} } \right]$	^58,61^	P_N_	900 W
				${\text{R}}_{{\text{S}}}$	0.49 Ω
				${\text{L}}_{{\text{S}}}$	0.0016 H
				P	5
				${\Phi }_{{\text{f}}}$	0.148 Wb
				R_Tb_	1.05 m
				J	0.016 kg/m^2^
Batteries		$$\left\{ {\begin{array}{*{20}c} {{\text{V}}_{{{\text{Batt}}}} = {\text{E}}_{0} - {\text{R}}_{{{\text{Batt}}}} .{\text{I}}_{{{\text{Batt}}}} - {\text{k}}.\smallint \left( {\frac{{{\text{I}}_{{{\text{Batt}}}} }}{{\text{Q}}}} \right).{\text{dt}}} \\ {{\text{SOC}} = 1 - \frac{{{\text{I}}_{{{\text{Batt}}}} .{\text{t}}}}{{{\text{C}}_{{{\text{Batt}}}} }}} \\ \end{array} } \right.$$	^37,61^	V_Batt_	12 V
				C_Batt_	100 Ah
				R_Batt_	0.795 Ω
				X_Batt_	0.07 Ω
				C_Batt_	44.96 mF
Supercapacities		$$\left\{ {\begin{array}{*{20}c} {{\text{U}}_{{{\text{sc}}}} = {\text{N}}_{{{\text{sc}} - {\text{s}}}} .{\text{V}}_{{{\text{sc}}}} = {\text{N}}_{{{\text{sc}} - {\text{s}}}} .\left( {{\text{V}}_{1} + {\text{R}}_{1} .{\text{I}}_{{{\text{sc}}}} } \right)} \\ { = {\text{N}}_{{{\text{sc}} - {\text{s}}}} .\left( {{\text{V}}_{1} + {\text{R}}_{1} .\frac{{{\text{i}}_{{{\text{sc}}}} }}{{{\text{N}}_{{{\text{sc}} - {\text{p}}}} }}} \right)} \\ \end{array} } \right.$$ ${\text{V}}_{2} = \frac{1}{{{\text{C}}_{2} }}\smallint {\text{i}}_{2} \left( {\text{t}} \right).{\text{dt}}\frac{1}{{{\text{C}}_{2} }}\smallint \frac{1}{{{\text{R}}_{2} }}\left( {{\text{v}}_{1} - {\text{v}}_{2} } \right).{\text{dt}}$ ${\text{Q}}_{2} = \smallint {\text{i}}_{2} \left( {\text{t}} \right).{\text{dt}}$ ${\text{i}}_{1} = {\text{i}}_{{{\text{sc}}}} - {\text{i}}_{2}$	^60,61^	C_N_	165 F
				ESR_DC_	60 m Ω
				IR_DC_	100A
				V_N_	48 V
				E_sc_	53 Wh
				V_max_	51 V
				I_max_	1900 A
				V_series_	750 V
				C_cells_	3000 F
				E_sc-cell_	3.0 Wh
				N_cells_	18

**Figure 3:**
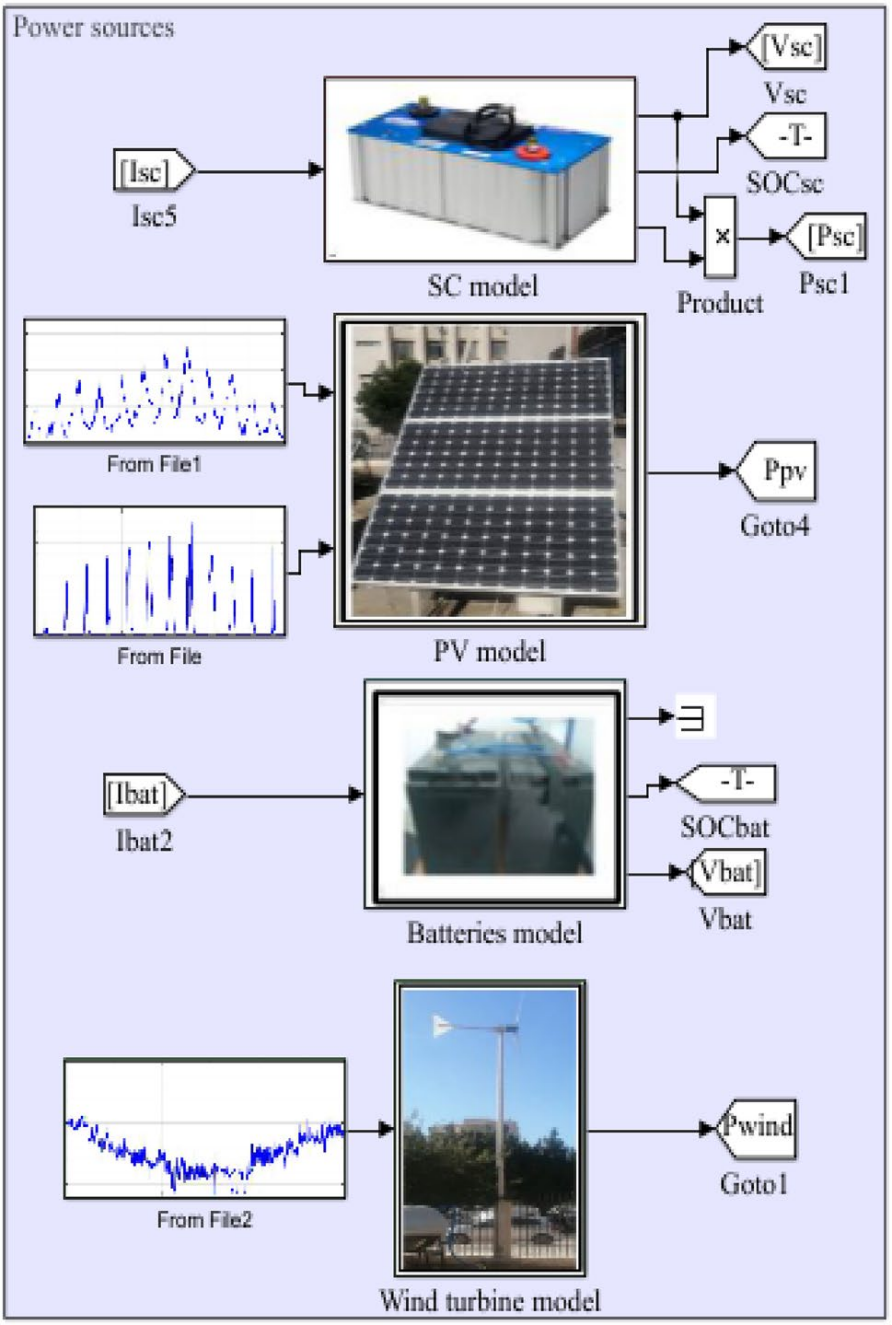
Simulink modeling of different power components.

**Figure 4:**
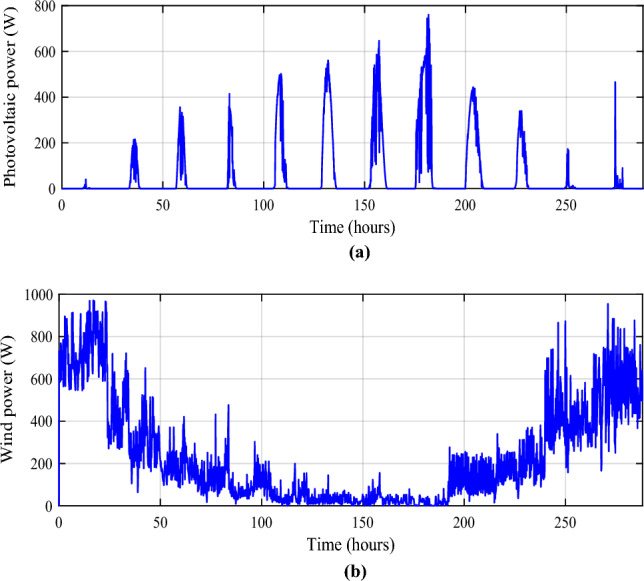
Obtained powers (PV and wind turbine) during a year. (**a**) Photovoltaic power. (**b**) Wind turbine power.

The different sources have been simulated under MATLAB/Simulink (Fig. 3) and the obtained powers are represented in Fig. 4a, b.

## Optimization of photovoltaic and wind generators

### Photovoltaic generator optimization

**Figure 5:**
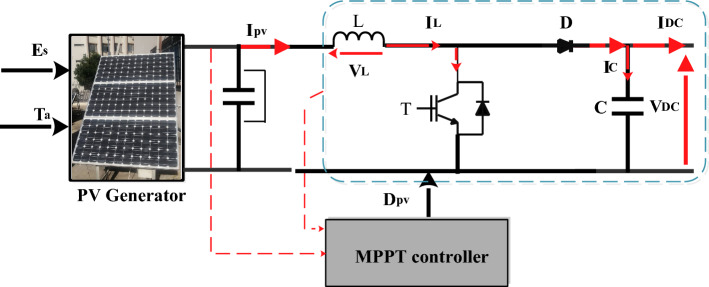
PV system with MPPT controller.

A boost converter’s main feature is its capacity to step up the input voltage, which makes it helpful in situations that require a higher voltage than what is available from the input source (Fig. 5). The electrical equations are:

1 $$\left\{ {\begin{array}{*{20}c} {V_{pv} = L\frac{{dI_{L} }}{dt} + \left( {1 - D_{pv} } \right)V_{dc} } \\ {\left( {1 - D_{pv} } \right)I_{L} = C\frac{{dV_{dc} }}{dt} + I_{dc} } \\ \end{array} } \right.$$

Then, it is obtained:

2 $$\left\{ {\begin{array}{*{20}c} {V_{dc} = \frac{1}{{\left( {1 - D_{pv} } \right)}}V_{pv} } \\ {I_{dc} = \left( {1 - D_{pv} } \right)I_{L} } \\ \end{array} } \right.$$

### Wind turbine optimization

**Figure 6:**
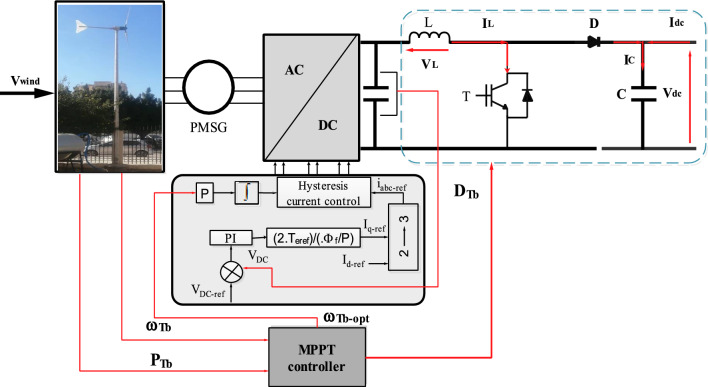
Wind turbine system with MPPT controller.

One of the main goals of the control is to extract the most available power from variable wind speeds (Fig. 6). The rotational speed variation is related to finding the optimum power point through duty cycle adjustment in voltage, and electromagnetic torque^33,61^.

### MPPT controllers for PV and wind turbine

#### P&O algorithm

**Figure 7:**
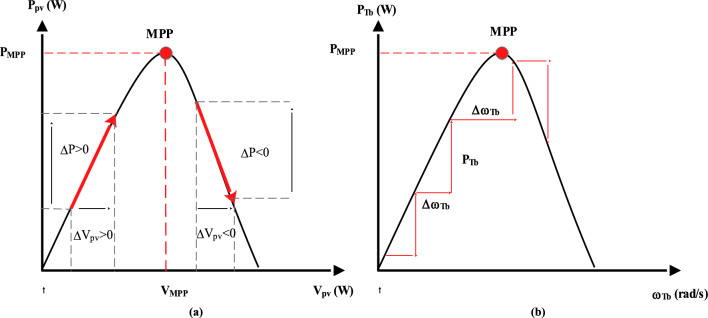
P&O algorithm principle. (**a**) Photovoltaic. (**b**) Wind turbine.

The P&O or Hill Climb Search (HCS) control is an extensively used MPPT method. The primary idea is to disturb the operating point of the solar panels or the wind turbine and then observe the subsequent change in power. The algorithm decides whether to increase or reduce the operating point based on this observation (Fig. 7)^8,12,15^.

The duty cycle is adjusted to find the maximum power point (MPP). It is perturbed by a small increment or decrement^75^.

3 $$\left\{ {\begin{array}{*{20}c} {{\text{D}}_{{{\text{PV}} - {\text{k}} + 1}} = {\text{ D}}_{{{\text{PV}} - {\text{k}}}} + {\text{K}}_{{{\text{dDpv}}}} \cdot {\text{sign}}\left( {\Delta {\text{P}}_{{{\text{pv}}}} } \right)} \\ {{\text{D}}_{{{\text{Tb}} - {\text{k}} + 1}} = {\text{ D}}_{{{\text{Tb}} - {\text{k}}}} + {\text{K}}_{{{\text{dDTb}}}} \cdot {\text{sign}}\left( {\Delta {\text{P}}_{{{\text{Tb}}}} } \right)} \\ \end{array} } \right.$$

where: ${\text{K}}_{{{\text{dDpv}}}}$ and ${\text{K}}_{{{\text{dDTb}}}}$ are proportionality constant, ΔP_pv_ and ΔP_Tb_ are is the change in PV and wind turbine power after perturbation, Sign (ΔP_pv_) and Sign (ΔP_Tb_) are the sign functions, indicating the direction of the change in PV and wind turbine power.

If the power elevates after the perturbation, it means that the MPP is pointing in the direction of the perturbation, and the duty cycle will be modified accordingly. And if the power decreases after the perturbation, it means that the MPP is in the opposite direction of the perturbation, and the duty cycle is modified accordingly.

#### FLC method

**Figure 8:**
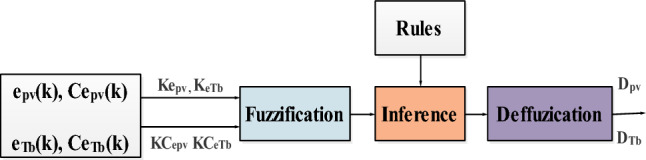
FLC block diagram.

Fuzzy logic controllers are very used in MPP research^9–12^. The MPPT method from FLC is an intelligent control approach used in PV and wind turbine systems to efficiently track and maintain the MPP of a solar array^75^. Fuzzy logic controllers use linguistic variables and rules to make decisions, making them well-suited for systems with uncertainties and non-linearities. The system consists of a block for calculating the variation of the error over time (Ce_pv_(k) or Ce_Tb_(k)), scaling factors associated with the error, its variation and the control variation (dD_pv_ ou dD_Tb_), fuzzy controller rules (Inference) and a defuzzification block used to convert control variation (Fig. 8).

This law is a function of the error and its variation (D_pv_ = ƒ(e_pv_, Ce_pv_),or D_Tb_ = ƒ(e_Tb_, Ce_Tb_). Consequently, activating the set of associated decision rules gives the variation in control dD (dD_pv_ or dD_Tb_) required, enabling the adjustment of such a control D (D_pv_ or D_Tb_).

The control law is as follow:

4 $$\left\{ {\begin{array}{*{20}c} {{\text{D}}_{{{\text{pv}} - {\text{K}} + 1}} = {\text{D}}_{{{\text{pv}} - {\text{K}}}} + {\text{K}}_{{{\text{dDpv}}}} { }.{\text{dD}}_{{{\text{pv}} - {\text{K}} + 1}} } \\ {{\text{D}}_{{{\text{Tb}} - {\text{K}} + 1}} = {\text{D}}_{{{\text{Tb}} - {\text{K}}}} + {\text{K}}_{{{\text{dDTb}}}} { }.{\text{dD}}_{{{\text{Tb}} - {\text{K}} + 1}} } \\ \end{array} } \right.$$

The calculation steps for the various controls are as follows^67^:

Calculation of the error:

5 $$\left\{ {\begin{array}{*{20}c} {{\text{e}}_{{{\text{pv}}}} \left( {\text{k}} \right) = \frac{{{\text{P}}_{{{\text{pv}}}} \left( {{\text{k}} + 1} \right) - {\text{P}}_{{{\text{pv}}}} \left( {\text{k}} \right)}}{{{\text{V}}_{{{\text{pv}}}} \left( {{\text{k}} + 1} \right) - {\text{V}}_{{{\text{pv}}}} \left( {\text{k}} \right)}}} \\ {{\text{e}}_{{{\text{Tb}}}} \left( {\text{k}} \right) = \frac{{{\text{P}}_{{{\text{Tb}}}} \left( {{\text{k}} + 1} \right) - {\text{P}}_{{{\text{Tb}}}} \left( {\text{k}} \right)}}{{{\upomega }_{{{\text{Tb}}}} \left( {{\text{k}} + 1} \right) - {\upomega }_{{{\text{Tb}}}} \left( {\text{k}} \right)}}} \\ \end{array} } \right.$$

Calculation of the variation of this error:

6 $$\left\{ {\begin{array}{*{20}c} {{\text{Ce}}_{{{\text{pv}}}} \left( {\text{k}} \right) = {\text{e}}_{{{\text{pv}}}} \left( {{\text{k}} + 1} \right) - {\text{e}}_{{{\text{pv}}}} \left( {\text{k}} \right)} \\ {{\text{Ce}}_{{{\text{Tb}}}} \left( {\text{k}} \right) = {\text{e}}_{{{\text{Tb}}}} \left( {{\text{k}} + 1} \right) - {\text{e}}_{{{\text{Tb}}}} \left( {\text{k}} \right)} \\ \end{array} } \right.$$

Calculation of the normalized values of e_pv_(k), e_Tb_(k), and Ce_pv_(k), Ce_Tb_(k), by:

7 $$\left\{ {\begin{array}{*{20}c} {{\text{X}}_{{{\text{epv}}}} = {\text{K}}_{{{\text{epv}}}} *{\text{e}}_{{{\text{pv}}}} } \\ {{\text{X}}_{{{\text{Cepv}}}} = {\text{K}}_{{{\text{Cepv}}}} *{\text{C}}_{{{\text{epv}}}} } \\ \end{array} } \right.$$

8 $$\left\{ {\begin{array}{*{20}c} {{\text{X}}_{{{\text{eTb}}}} = {\text{K}}_{{{\text{eTb}}}} *{\text{e}}_{{{\text{Tb}}}} } \\ {{\text{X}}_{{{\text{CeTb}}}} = {\text{K}}_{{{\text{CeTb}}}} *{\text{C}}_{{{\text{eTb}}}} } \\ \end{array} } \right.$$

where ${\text{K}}_{{{\text{epv}}}} ,{\text{ K}}_{{{\text{eTb}}}}$ and ${\text{K}}_{{{\text{Cepv}}}} ,{\text{ K}}_{{{\text{CeTb}}}}$ are the scaling factors.

**Figure 9:**
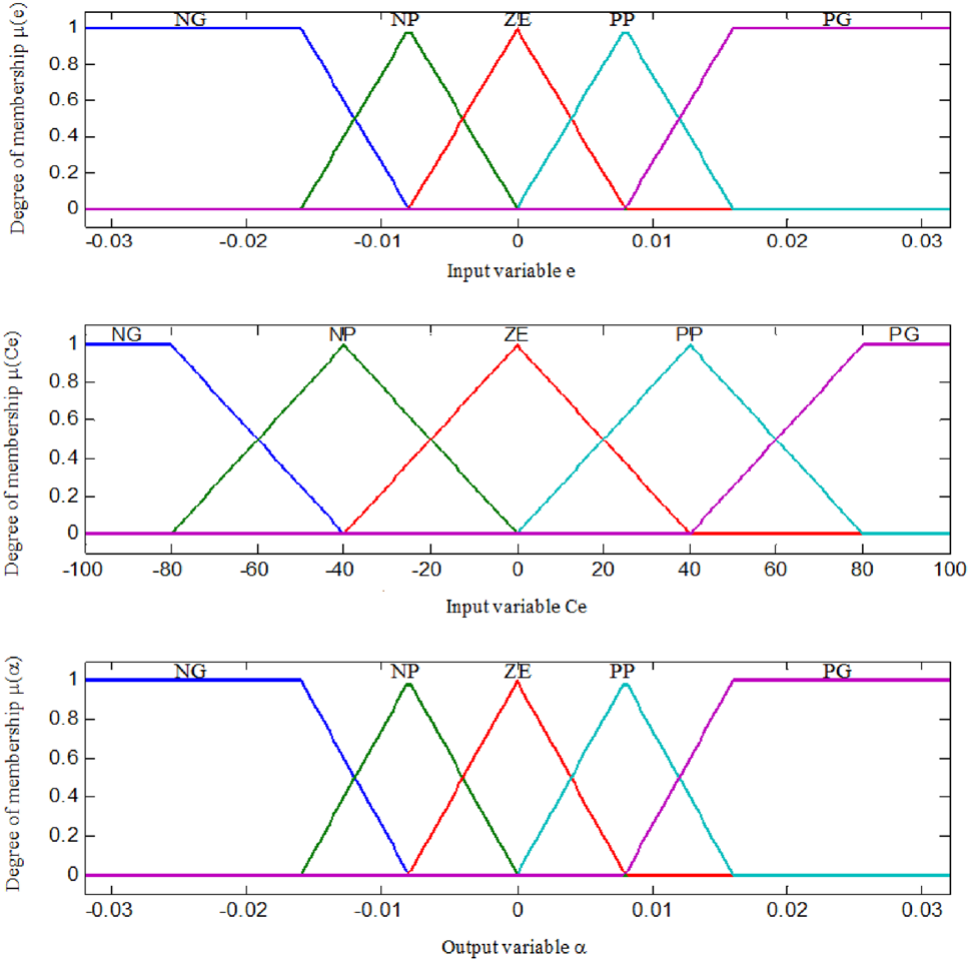
Membership functions for input variable e, input variable Ce and output variable.

The purpose of the fuzzification process is to introduce fuzzy sets of required values with a certain degree of membership. The defined classes are (Fig. 9): NB: Negative Large, NS: Negative Small, ZE: Zero Environment, PB: Positive Large, and PS: Positive Small. Defuzzification is the last step of the FLC method.

Fuzzy rules are utilized to compute the controller output signal based on the input signals (Table 4).

**Table 4:** FLC rules.

e_pv_, eTb	NG	NP	ZE	PP	PG
Ce_pv_, Ce_pv_					
NG	ZE	ZE	PG	PG	PG
NP	ZE	EZ	PP	PP	PP
ZE	PP	ZE	ZE	ZE	NP
PP	NP	NP	NP	ZE	ZE
PG	NG	NG	NG	ZE	ZE

The center of gravity becomes:

9 $$\left\{ {\begin{array}{*{20}c} {{\text{D}}_{{{\text{pV}}}} = { }\frac{{\mathop \sum \nolimits_{{{\text{i}} = 1}}^{{\text{n}}} {\upmu }\left( {{\text{D}}_{{{\text{PV}} - {\text{i}}}} } \right) - {\text{D}}_{{{\text{PV}}}} }}{{\mathop \sum \nolimits_{{{\text{i}} = 1}}^{{\text{n}}} {\upmu }\left( {{\text{D}}_{{{\text{PV}} - {\text{i}}}} } \right)}}} \\ {{\text{D}}_{{{\text{Tb}}}} = { }\frac{{\mathop \sum \nolimits_{{{\text{i}} = 1}}^{{\text{n}}} {\upmu }\left( {{\text{D}}_{{{\text{Tb}} - {\text{i}}}} } \right) - {\text{D}}_{{{\text{Tb}}}} }}{{\mathop \sum \nolimits_{{{\text{i}} = 1}}^{{\text{n}}} {\upmu }\left( {{\text{D}}_{{{\text{Tb}} - {\text{i}}}} } \right)}}} \\ \end{array} } \right.$$

with μ(D_pv−i_), μ(D_Tb-i_) are the degree of activation of the ith rule and D_pv_, D_Tb_ are the centroid abscissa of the ith class.

#### Proposed HPV (P&O/FLC)

**Figure 10:**
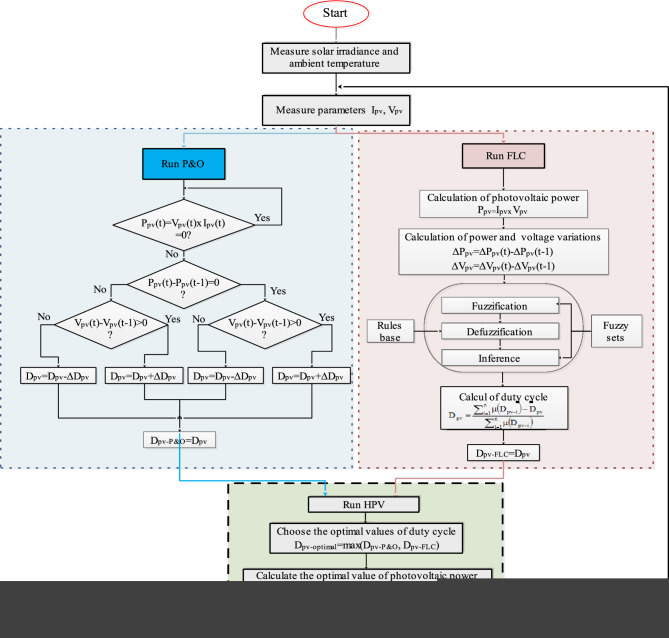
Flowchart of HPV (P&O/FLC).

The proposed strategy concerns the hybridization of P&O and FLC algorithms. First, the PV voltage, current and duty cycle (D_pv_) of each MPPT strategy is calculated. In the second step, the optimal duty cycle (D_pv-optimal_) is deduced (D_optimal_ = max(D_PV-P&O_, D_pv_-_FLC_)) and applied in the HPV (P&O/FLC) method. The proposed flowchart is given in Fig. 10.

In this comparative analysis section of our paper, we have two prominent methods for optimizing photovoltaic system performance: Perturb and Observe MPPT and Fuzzy Logic MPPT. The Table 5 below provides a succinct overview of their respective principles, advantages, and drawbacks, offering a valuable qualitative comparison and useful insights into the applicability of each technique in maximizing power output from PV panels.

**Table 5:** Comparative analysis study of the studied MPPT methods.

Aspect	Perturb and Observe MPPT	FLC-MPPT
Operation principle	Adjusts PV voltage and measures power	Uses fuzzy logic control to track the MPP
Advantages	Simple implementation	Robustness against variations
	Widely used	Good performance in partial shading
	Fast convergence	Efficient in uncertain environments
Drawbacks	Oscillations around the MPP	Complexity in implementation
	Susceptible to local minimas	Requires more computational resources
	Sensitive to temperaturevariations	Choice of system rules

**Figure 11:**
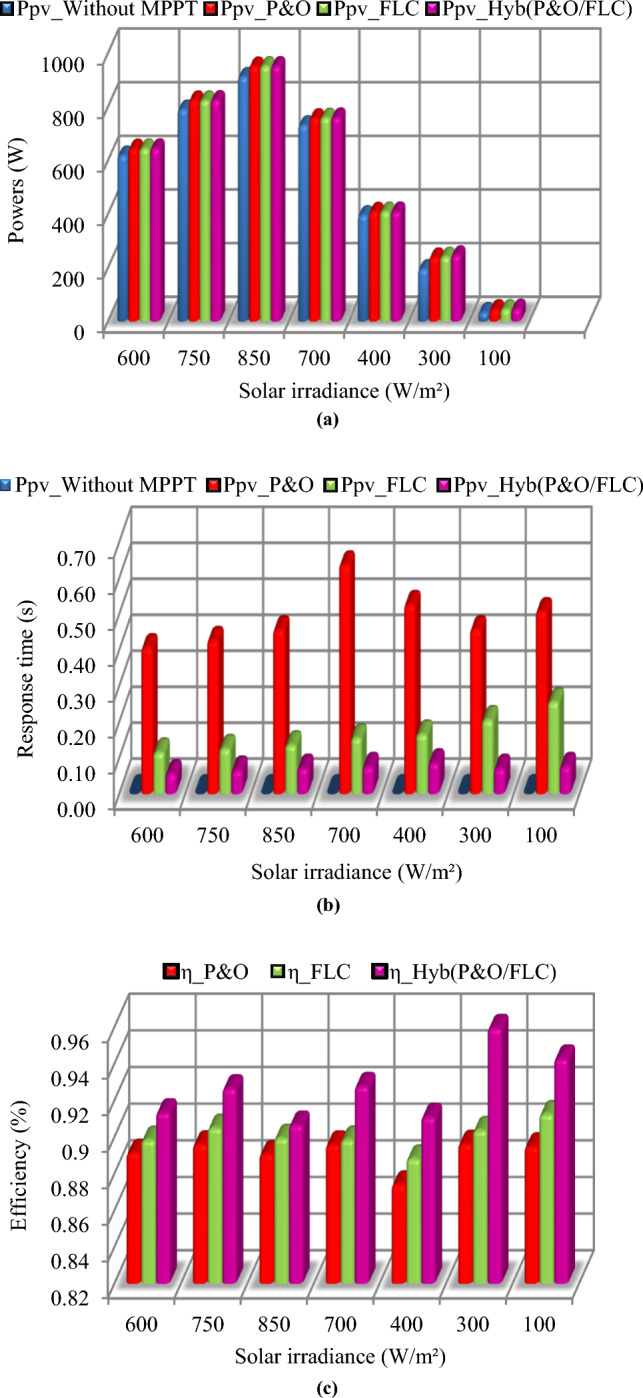
Comparative of the three MPPT methods in PV. (**a**) in terms of powers. (**b**) in terms of response time. (**c**) in terms of efficiency.

A comparison between the three MPPT methods in photovoltaic system has been made in terms of maximum power, response time and efficiency (Fig. 11). It is noticed that the Hybrid (P&O/FLC) allows us to obtain a fast response since it reaches its optimal value rapidly compared to the P&O and FLC methods which require a more time to follow the MPP). The hybrid (P&O/FLC) reduce not only the convergence time to follow the MPP, but also decreases the steady-state power oscillation.

The photovoltaic power gain between the different methods can be written as following.

10 $$\left\{ {\begin{array}{*{20}c} {{\text{DP}}_{{{\text{pv}}\left( {{\text{Hpv }}/{\text{P}}\& O} \right)}} = {\text{P}}_{{{\text{pv}} - {\text{Hpv}}}} - {\text{P}}_{{{\text{pv}} - {\text{P}}\& O}} } \\ {{\text{DP}}_{{{\text{pv}}\left( {{\text{Hpv }}/{\text{FLC}}} \right){ }}} = {\text{P}}_{{{\text{pv}} - {\text{Hpv}}}} - {\text{P}}_{{{\text{pv}} - {\text{FLC}}}} } \\ {{\text{DP}}_{{{\text{pv}}\left( {{\text{FLC}}/{\text{P}}\& O} \right) }} = {\text{P}}_{{{\text{pv}} - {\text{FLC}}}} - {\text{P}}_{{{\text{pv}} - {\text{P}}\& O}} } \\ \end{array} } \right.$$

**Figure 12:**
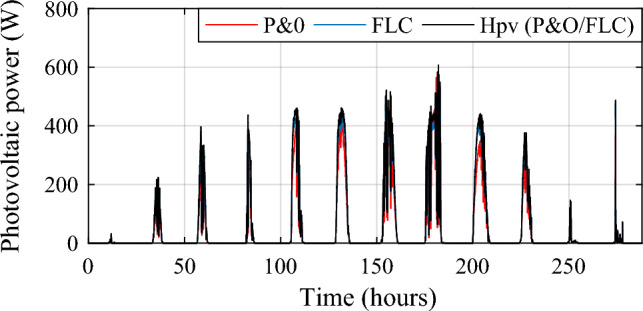
Optimized Photovoltaic power under three MPPT methods.

**Figure 13:**
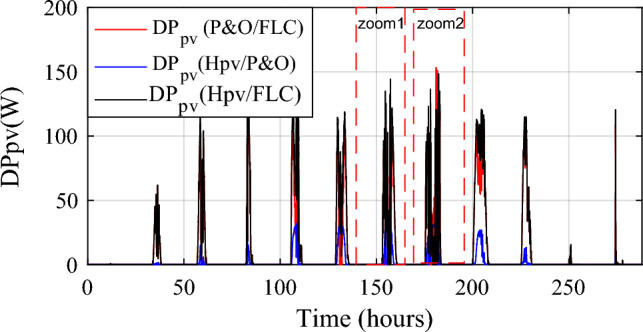
Photovoltaic power gain using the different MPPT strategies.

**Figure 14:**
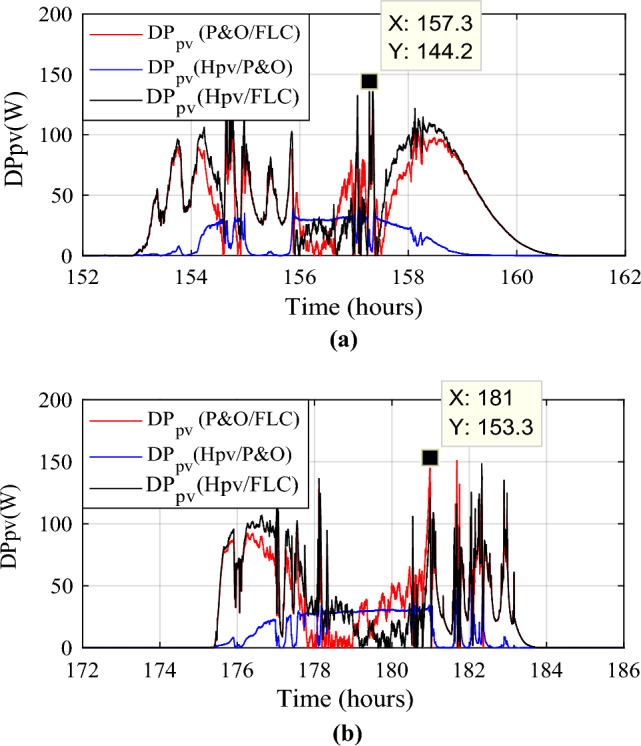
Zooms on photovoltaic power gain using the different MPPT strategies. (**a**) Zoom1. (**b**) Zoom2.

The PV power obtained under the three MPPTs is shown in Fig. 12 and obtained PV gain power is represented in Fig. 13. Two different zooms have been made to show the different gains obtained between the proposed hybrid MPPT and the no-hybrid ones (Fig. 14a, b).

The power gain between the suggested hybrid approach Hpv and the P&O strategy can reach 144.2 W (black color), as shown in Fig. 14a. And between P&O and FLC (Fig. 14b), it is acquired a power up to 153.3 W (in red color). The hybrid MPPT strategy (Hpv (P&O/FLC)) outperforms the non-hybrid methods regardless of wind speed variations.

**Figure 15:**
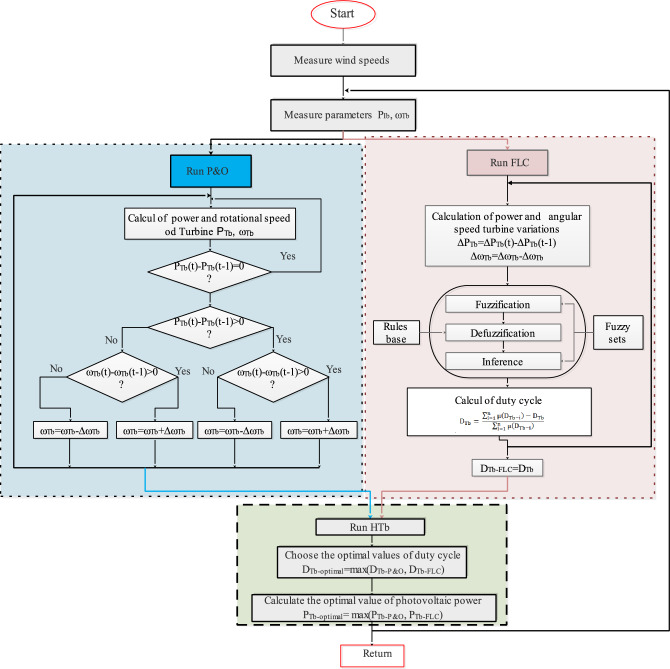
Flowchart of power optimization calculation.

In the present work, for wind turbine optimization, two approaches (P&O and FLC) were chosen to be combined. This optimization strategy is provided to achieve better results. The first stage allows us to choose distinct ideal values for each MPPT method, while the second stage calculates the optimal rotational speed and electromagnetic torque values. In the third stage, the optimal turbine power is obtained. The proposed optimized power calculation is presented in the flowchart below (Fig. 15).

**Figure 16:**
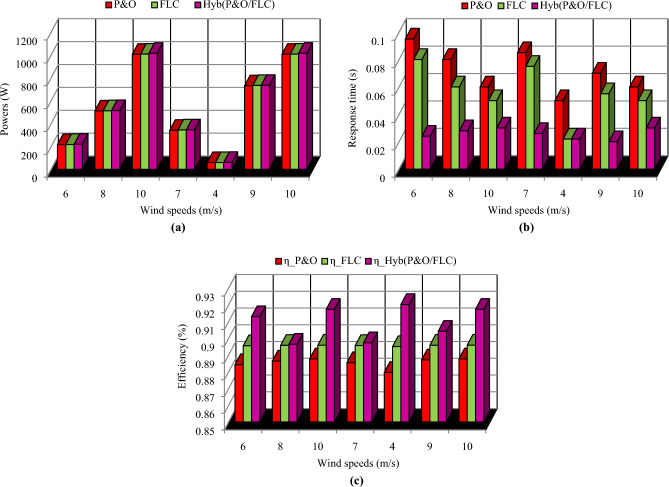
Comparative study of the three MPPT methods in wind turbine. (**a**) in terms of powers. (**b**) in terms of response time. (**c**) in terms of efficiency.

A comparison between the three MPPT methods in wind turbine system has been made in terms of maximum power, response time and efficiency (Fig. 16).

The hybrid (P&O/FLC) method provides the best results in PV system and wind turbine, therefore, it is the selected MPPT method used each generator (PV and wind) of the studied system.

**Figure 17:**
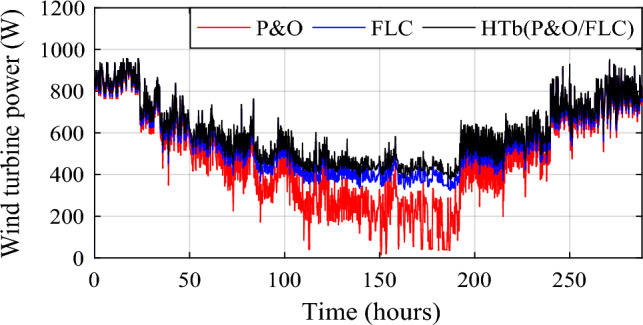
Optimized wind turbine power under three MPPT methods.

The wind turbine power obtained under the three MPPTs is shown in Fig. 17. The wind power gain between the different methods can be written as:

11 $$\left\{ {\begin{array}{*{20}c} {{\text{DP}}_{{{\text{wind}}\left( {{\text{HTb}}/{\text{P}}\& {\text{O}}} \right)}} = {\text{P}}_{{{\text{wind}} - {\text{HTb}}}} - {\text{P}}_{{{\text{wind}} - {\text{P}}\& {\text{O}}}} } \\ {{\text{DP}}_{{{\text{wind}}\left( {{\text{HTb}}/{\text{FLC}}} \right){ }}} = {\text{P}}_{{{\text{wind}} - {\text{HTb}}}} - {\text{P}}_{{{\text{wind}} - {\text{FLC}}}} } \\ {{\text{DP}}_{{{\text{wind}}({\text{FLC}}/{\text{P}}\& {\text{O}}}} ) = {\text{P}}_{{{\text{wind}} - {\text{FLC}}}} - {\text{P}}_{{{\text{wind}} - {\text{P}}\& {\text{O}}}} } \\ \end{array} } \right.$$

**Figure 18:**
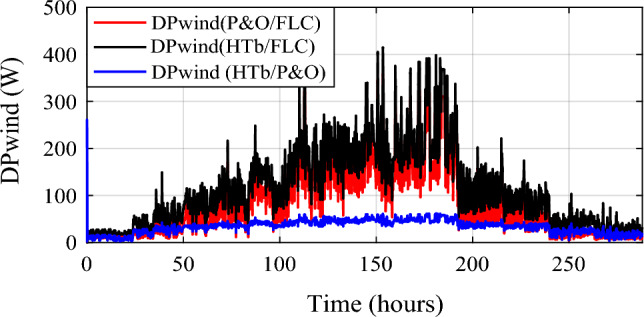
Wind power gain using the different MPPT strategies.

The obtained wind gain power is represented in Fig. 18. Two different zooms have been made to show the different gains obtained between the proposed hybrid MPPT and the no-hybrid ones.

**Figure 19:**
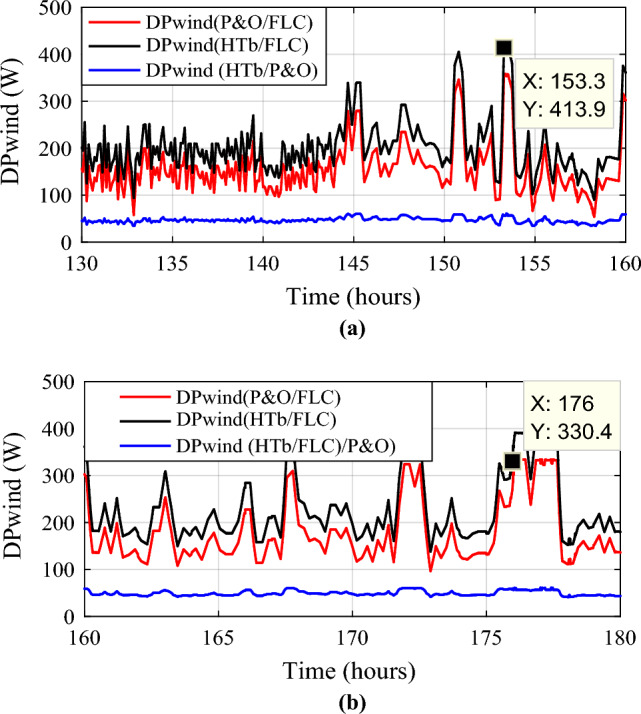
Zooms on wind power gain using the different MPPT strategies. (**a**) Zoom1. (**b**) Zoom2.

**Figure 20:**
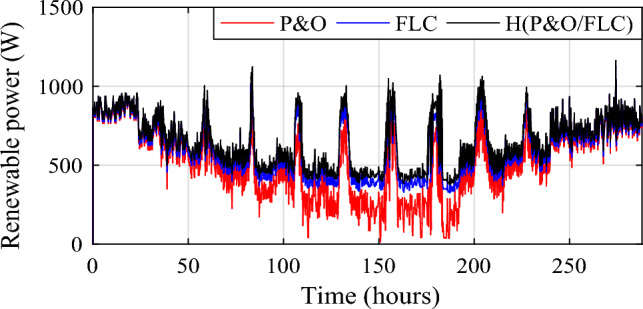
Optimized renewable hybrid power under three MPPT methods.

**Figure 21:**
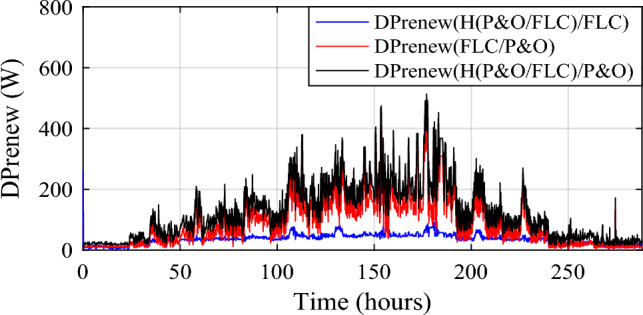
Renewable power gain using the different MPPT strategies.

Wind power gain using the different MPPT strategies are shown in Fig. 19, and zooms on wind power gain using the different MPPT strategies are given in Fig. 20a, b. In Fig. 20a, power gain between the proposed hybrid method HTb and P&O strategy can reach up to 413.9 W (black color), and between P&O and FLC (Fig. 20b), it is acquired a power up to 330.4 W (in red color). The renewable power is the total of the PV and wind turbine capacities (Fig. 21).

12 $${\text{P}}_{{{\text{Re}}\;{\text{new}}}} = P_{{{\text{pv}} - {\text{optimal}}}} + P_{{{\text{wind}} - {\text{optimal}}}}$$

13 $$\left\{ {\begin{array}{*{20}c} {{\text{DP}}_{{{\text{renew}}\left( {{\text{P}}\& {\text{O}}/{\text{FLC}}} \right){ }/\left( {{\text{P}}\& {\text{O}}} \right){ }}} = {\text{P}}_{{{\text{renew}} - {\text{H}}\left( {{\text{P}}\& {\text{O}}/{\text{FLC}}} \right)}} - {\text{P}}_{{{\text{renew}} - {\text{P}}\& {\text{O}}}} } \\ {{\text{DP}}_{{{\text{renew}}({\text{H}}\left( {{\text{P}}\& {\text{O}}/{\text{FLC}}} \right)\left( {{\text{P}}\& {\text{O}}} \right){ }}} = {\text{P}}_{{{\text{renew}} - {\text{H}}\left( {{\text{P}}\& {\text{O}}/{\text{FLC}}} \right)}} - {\text{P}}_{{{\text{renew}} - {\text{FLC}}}} } \\ {{\text{DP}}_{{{\text{renew}}({\text{P}}\& {\text{O}}/{\text{FLC}}}} ) = {\text{P}}_{{{\text{renew}} - {\text{FLC}}}} - {\text{P}}_{{{\text{renew}} - {\text{P}}\& {\text{O}}}} } \\ \end{array} } \right.$$

**Figure 22:**
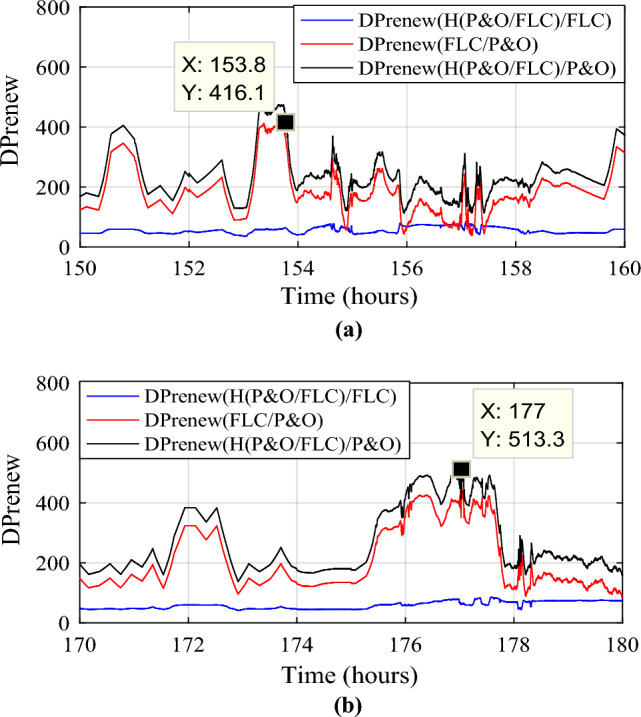
Zooms on renewable power gain using the different MPPT strategies. (**a**) Zoom1. (**b**) Zoom2.

The renewable power gain is represented in Fig. 22. Two different zooms have been made to show the different gains obtained between the proposed hybrid MPPT and the no-hybrid ones (Fig. 22a, b). It is noticed that power gain obtained due to the savings in wind and PV power. The power increase between the suggested hybrid approach and the P&O strategy (in black) was 513.3 W, while the maximum power gain throughout FLC and P&O was 416.3 W (in red).

The same conclusions for the hybrid power, significant power gains are obtained due to the savings in wind and PV power. Power gain between the proposed hybrid method and the P&O strategy (in black color) has reached a value of 513.3 W and between FLC and P&Oit has attained a maximum power gain of 416.3 W (in red color).

## Proposed hybrid energy storage system

**Figure 23:**
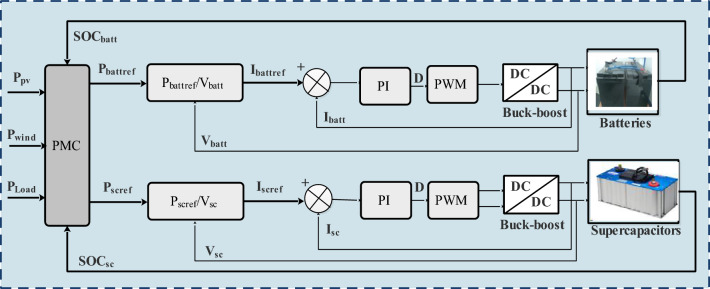
Proposed Hybrid Energy storage system.

**Figure 24:**
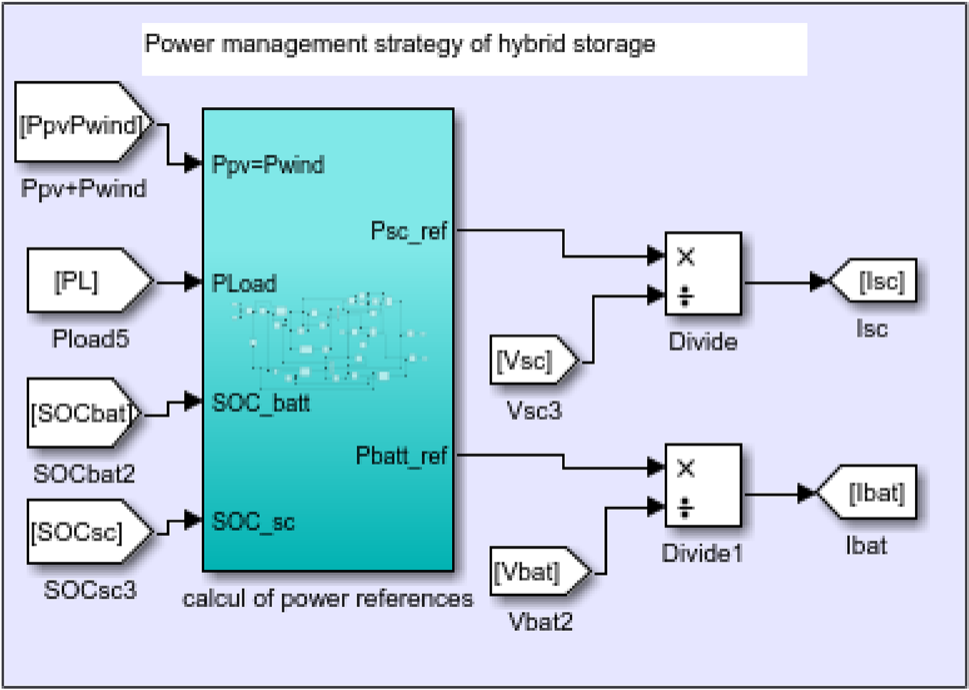
Determination of batteries and SCs currents.

In an isolated PV/wind turbine system, batteries play a crucial role due to several specific benefits and reasons. Furthermore, they are essential for storing and managing energy, ensuring a reliable and continuous power supply. Unfortunately, their energy density is still relatively lower compared to some other forms of energy storage. Moreover, they have a limited number of charge–discharge cycles before their capacity degrades significantly^54^. Supercapacitors (SCs) offer distinct advantages in certain applications. However, their limitations, such as low energy density and specific voltage needs, make them most useful when paired with other energy storage technologies, such as batteries (Fig. 23). To determinate the batteries and SCs currents, the references powers have been calculated as shown in Fig. 24 under MATLAB/Simulink.

While integrating supercapacitors alongside batteries in an energy storage system offers several advantages, it also presents trade-offs and challenges that must be carefully managed to realize the full potential of the hybrid storage configuration. Balancing factors such as energy density, cost, system integration, control, and safety is crucial to designing an effective and reliable hybrid energy storage solution.

**Figure 25:**
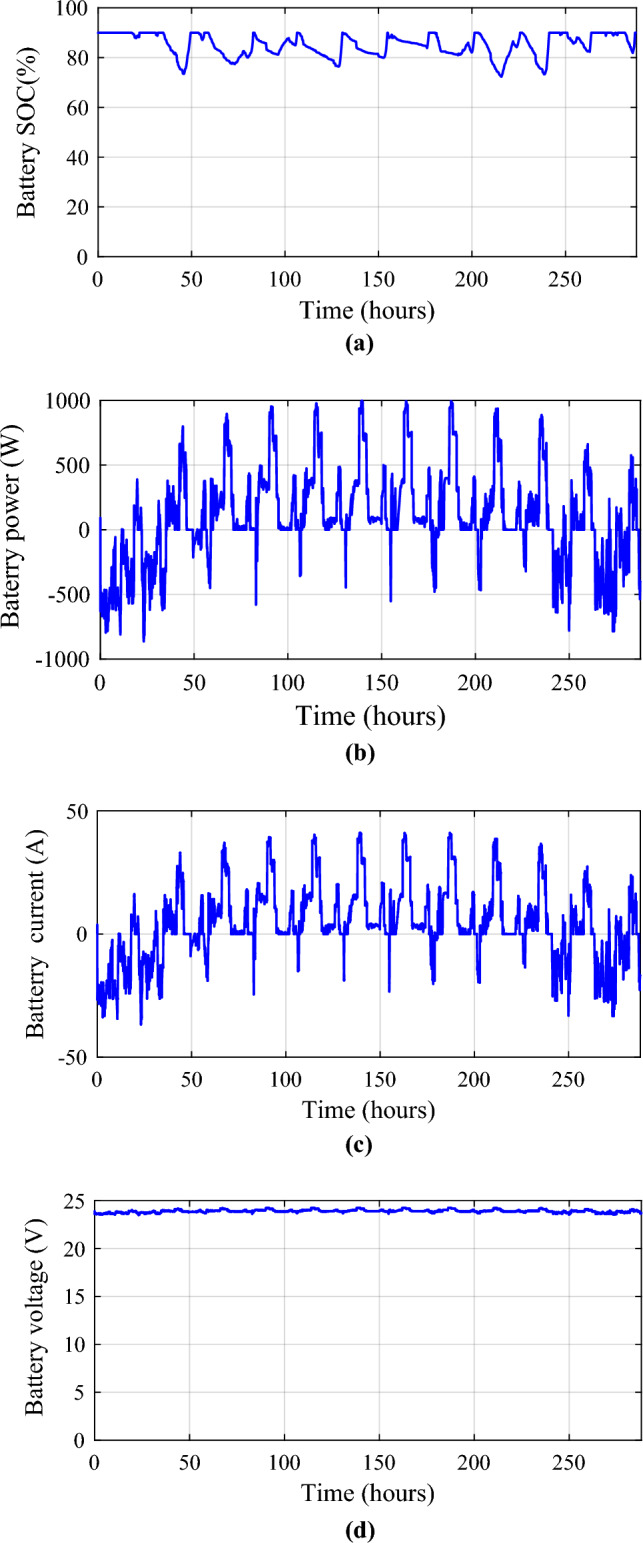
Battery performances. (**a**) SOC. (**b**) Battery power. (**c**) Battery current. (**d**) Voltage battery.

**Figure 26:**
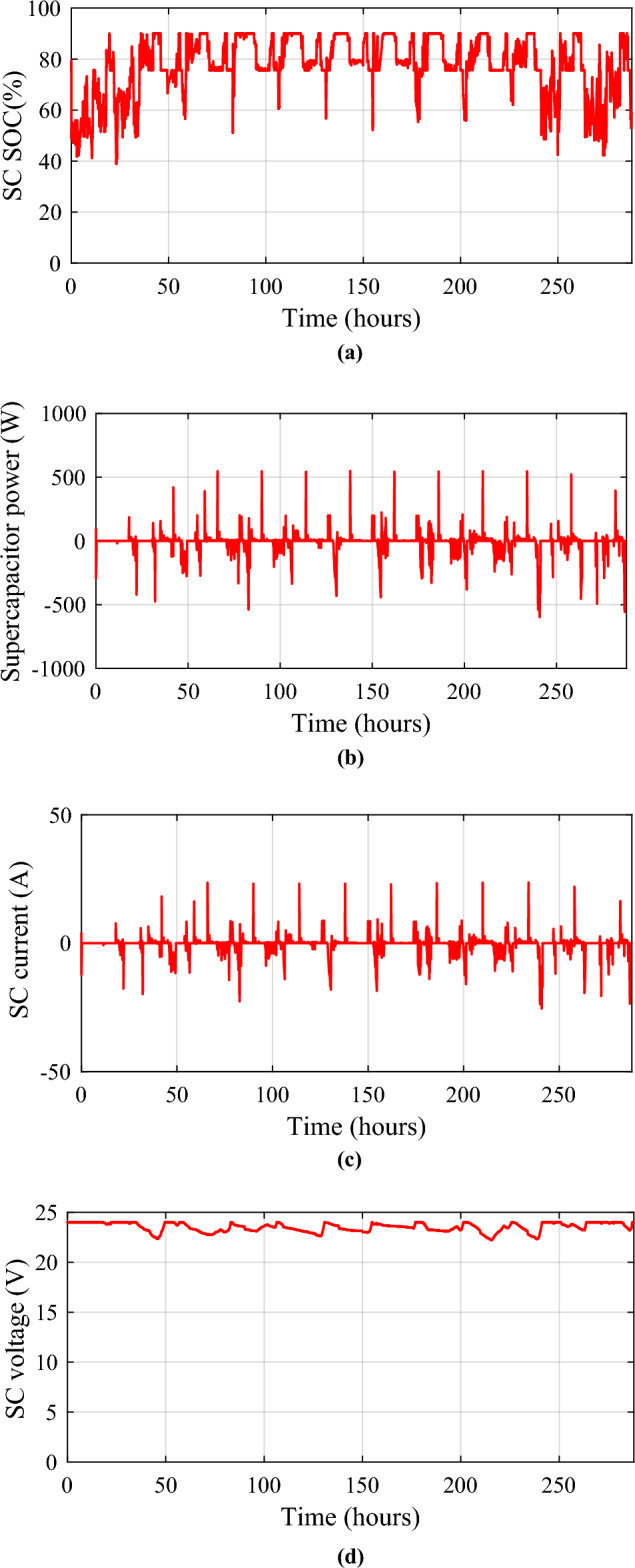
Supercapacities performances. (**a**) SC state of charge. (**b**) SC power. (**c**) SC current. (**d**) SC voltage.

Figures 25 and 26 show the differences in battery and SCs performance in terms of SOC, power, current, and voltage. The voltage of the batteries and SCs fluctuates with the amount of power absorbed/injected into the DC bus.

**Figure 27:**
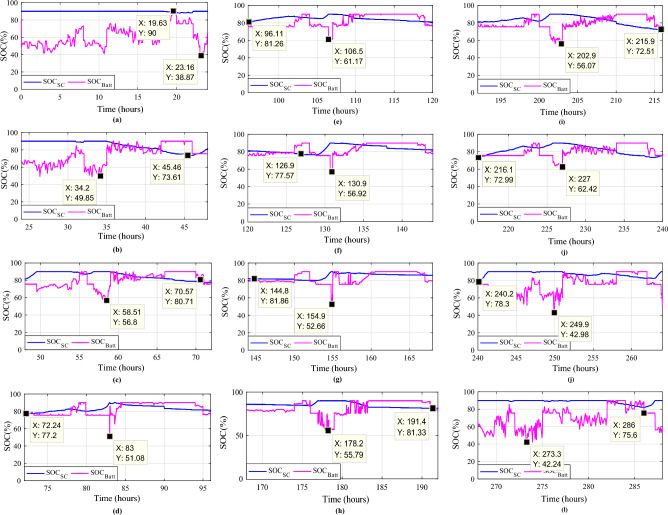
Battery and supercapacitors state of charge for the different profiles. (**a**) Profile 1. (**b**) Profile 2. (**c**) Profile 3. (**d**) Profile 4. (**e**) Profile 5. (**f**) Profile 6. (**g**) Profile 7. (**h**) Profile 8. (**i**) Profile 9. (**j**) Profile 10. (**k**) Profile 11. (**l**) Profile 12.

Monitoring these voltage profiles is vital for making certain the energy storage components are correctly charged and discharged. The batteries and SCs SOCs are depicted concurrently for each day (Fig. 27) to examine their fluctuations. The battery’s state of charge (SOC) is appropriately controlled and maintained at 72.99% (in October) and 90%, but the supercapacitor’s SOC ranges from 38.87 (in January) to 90%. Regardless of fluctuations in PV, wind turbine, and load power profiles, the SOCs of the batteries and SCs remain within acceptable limits.

## Proposed PMC of optimized PV/wind turbine system HESS

The system’s operation is dependent on power availability and the dynamics of needs comprising the PV, wind turbine, and the load. The two storage components (batteries and SCs), possess the capability to operate in both charge and discharge scenarios. This flexibility allows them to adapt to the varying power needs of the system.A prolonged power imbalance within the PV/wind turbine/storage system isc onsidered a potential problem. Such imbalances could have negative consequences, including deep discharge oover loading of the storage system.

We have:

14 $$P_{Loadcalc} = P_{pv - optimal} + P_{wind - optimal} + P_{Batt} + P_{SC}$$

15 $$\Delta P = P_{Load} - \left( {P_{pv - optimal} + P_{wind - optimal} } \right)$$

**Figure 28:**
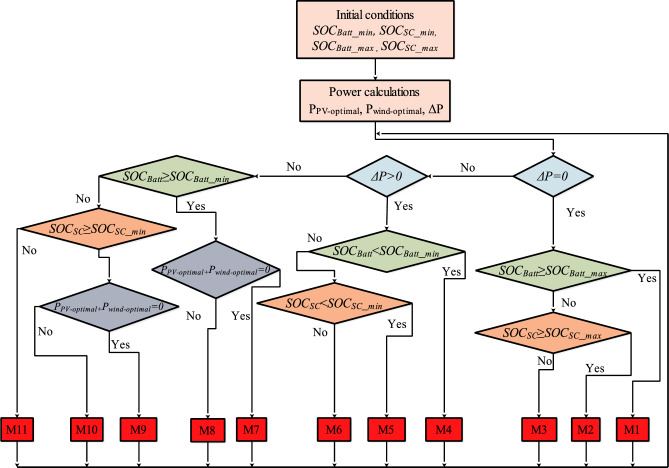
PMC flowchart of Photovoltaic/wind turbine with storage.

The studied system is described to operate under eleven distinct modes (Table 6). These modes encompass various scenarios. The full-load scenario refers to a state in which the energy storage components have reached their maximum load capacity, while the normal charge/discharge scenario suggests a regular, balanced energy flow within the system. Whereas, the transient scenario involves the system’s ability to effectively manage sudden or transitory changes in energy dynamics. Figure 28 depicts the flowchart of the proposed PMC for a PV/wind turbine system with hybrid storage.

**Table 6:** The different established modes and scenarios.

Cases	Equations	Scenarios
Mode 1 (M1)	$\Delta {\text{P}} = 0$ ${\text{P}}_{{{\text{Load}}}} = {\text{P}}_{{{\text{pv}} - {\text{optimal}}}} + {\text{P}}_{{{\text{wind}} - {\text{optimal}}}}$ ${\text{SOC}}_{{{\text{Batt}}}} \ge {\text{SOC}}_{{{\text{Batt}}\_{\text{max}}}}$	
Mode 2 (M2)	$\Delta {\text{P}} = 0$ ${\text{P}}_{{{\text{Load}}}} = {\text{P}}_{{{\text{pv}} - {\text{optimal}}}} + {\text{P}}_{{{\text{wind}} - {\text{optimal}}}}$ ${\text{SOC}}_{{{\text{SC}}}} \ge {\text{SOC}}_{{{\text{SC}}\_{\text{max}}}}$	
Mode 3 (M3)	$\Delta {\text{P}} = 0$ ${\text{P}}_{{L{\text{oad}}}} = {\text{P}}_{{{\text{pv}} - {\text{optimal}}}} + {\text{P}}_{{{\text{wind}} - {\text{optimal}}}}$ ${\text{SOC}}_{{{\text{Batt}}}} < {\text{SOC}}_{{{\text{Batt}}\_{\text{max}}}}$ ${\text{SOC}}_{{{\text{SC}}}} < {\text{SOC}}_{{{\text{SC}}\_{\text{max}}}}$	
Mode 4 (M4)	$\Delta {\text{P}} > 0$ ${\text{P}}_{{{\text{Load}}}} > {\text{P}}_{{{\text{pv}} - {\text{optimal}}}} + {\text{P}}_{{{\text{wind}} - {\text{optimal}}}}$ ${\text{SOC}}_{{{\text{Batt}}}} < {\text{SOC}}_{{{\text{Batt}}_{{{\text{min}}}} }}$ ${\text{P}}_{{{\text{Batt}}}} = {\text{P}}_{{{\text{load}}}} - {\text{P}}_{{{\text{pv}} - {\text{optimal}}}} - {\text{P}}_{{{\text{wind}} - {\text{optimal}}}}$	
Mode 5 (M5)	$\Delta {\text{P}} > 0$ ${\text{P}}_{{{\text{Load}}}} > {\text{P}}_{{{\text{pv}} - {\text{optimal}}}} + {\text{P}}_{{{\text{wind}} - {\text{optimal}}}}$ ${\text{SOC}}_{{{\text{SC}}}} < {\text{SOC}}_{{{\text{SC}}\_{\text{min}}}}$ ${\text{P}}_{{{\text{SC}}}} = {\text{P}}_{{{\text{load}}}} - {\text{P}}_{{{\text{pv}} - {\text{optimal}}}} - {\text{P}}_{{{\text{wind}} - {\text{optimal}}}}$	
Mode 6 (M6)	$\Delta {\text{P}} > 0$ ${\text{P}}_{Load} = {\text{P}}_{{{\text{pv}} - {\text{optimal}}}} + {\text{P}}_{{{\text{wind}} - {\text{optimal}}}}$ ${\text{SOC}}_{{{\text{Batt}}}} < {\text{SOC}}_{{{\text{Batt}}_{{{\text{min}}}} }}$ ${\text{SOC}}_{{{\text{SC}}}} < {\text{SOC}}_{{{\text{SC}}_{{{\text{min}}}} }}$ ${\text{P}}_{{{\text{Batt}}}} = {\text{P}}_{{{\text{load}}}} - {\text{P}}_{{{\text{pv}} - {\text{optimal}}}} - {\text{P}}_{{{\text{wind}} - {\text{optimal}}}} /2$ ${\text{P}}_{{{\text{SC}}}} = {\text{P}}_{{{\text{load}}}} - {\text{P}}_{{{\text{pv}} - {\text{optimal}}}} - {\text{P}}_{{{\text{wind}} - {\text{optimal}}}}$/2	
Mode 7 (M7)	$\Delta {\text{P}} < 0$ ${\text{P}}_{{{\text{pv}} - {\text{optimal}}}} + {\text{P}}_{{{\text{wind}} - {\text{optimal}}}} = 0$ ${\text{P}}_{{{\text{Load}}}} = {\text{P}}_{{{\text{Batt}}}}$ ${\text{SOC}}_{{{\text{Batt}}}} > {\text{SOC}}_{{{\text{Batt}}\_{\text{min}}}}$	
Mode 8 (M8)	$\Delta {\text{P}} < 0$ ${\text{P}}_{{{\text{pv}} - {\text{optimal}}}} + {\text{P}}_{{{\text{wind}} - {\text{optimal}}}} > 0$ ${\text{P}}_{{{\text{Load}}}} = {\text{P}}_{{{\text{pv}} - {\text{optimal}}}} + {\text{P}}_{{{\text{wind}} - {\text{optimal}}}} + {\text{P}}_{{{\text{Batt}}}}$ ${\text{SOC}}_{{{\text{Batt}}}} > {\text{SOC}}_{{{\text{Batt}}\_{\text{min}}}}$	
Mode 9 (M9)	$\Delta {\text{P}} < 0$ ${\text{P}}_{{{\text{pv}} - {\text{optimal}}}} + {\text{P}}_{{{\text{wind}} - {\text{optimal}}}} = 0$ ${\text{P}}_{{{\text{Load}}}} = {\text{P}}_{{{\text{SC}}}}$ ${\text{SOC}}_{{{\text{SC}}}} > {\text{SOC}}_{{{\text{SC}}\_{\text{min}}}}$	
Mode 10 (M10)	$\Delta {\text{P}} < 0$ ${\text{P}}_{{{\text{pv}} - {\text{optimal}}}} + {\text{P}}_{{{\text{wind}} - {\text{optimal}}}} > 0$ ${\text{P}}_{{{\text{Load}}}} = {\text{P}}_{{{\text{pv}} - {\text{optimal}}}} + {\text{P}}_{{{\text{wind}} - {\text{optimal}}}} + {\text{P}}_{{{\text{SC}}}}$ ${\text{SOC}}_{{{\text{SC}}}} > {\text{SOC}}_{{{\text{SC}}\_{\text{min}}}}$	
Mode 11 (M11)	$\Delta {\text{P}} < 0$ ${\text{P}}_{{{\text{pv}} - {\text{optimal}}}} + {\text{P}}_{{{\text{wind}} - {\text{optimal}}}} > 0$ ${\text{P}}_{{{\text{Load}}}} = 0$ ${\text{SOC}}_{{{\text{Batt}}}} \le {\text{SOC}}_{{{\text{Batt}}_{{{\text{min}}}} }}$ ${\text{SOC}}_{{{\text{SC}}}} \le {\text{SOC}}_{{{\text{SC}}\_{\text{min}}}}$

In Mode 1, the batteries are charged, so they are disconnected, while in Mode 2, the supercapacitors (SCs) are fully charged, leading to their disconnection. The algorithm operates in Mode 3 to avoid deep discharge, thus disconnecting both batteries and SCs. In Mode 4, solar and wind power generation supply the load, with excess power directed towards charging the batteries.

Similarly, in Mode 5, PV and wind power generated power the load, while excess power charges the SCs. In Mode 6, PV and wind turbines supply the load, and any extra power is used to charge both batteries and SCs.

Mode 7 utilizes charged batteries to supply the load, while Mode 8 involves charging the batteries when renewable power is not zero, with all sources supplying the load. Mode 9 sees the load fed by charged SCs, while in Mode 10, if SOC_SC_ > SOC_SC_min_, SCs compensate for the deficit of PV and wind power. Finally, in Mode 11, the load is not supplied.

The proposed power management controller strategy for reliable hybridization of multi-source systems using hybrid Maximum Power Point Tracking (MPPT) algorithms raises important considerations regarding computational complexity, real-time feasibility, scalability, and computational efficiency.

The computational complexity of the proposed power management controller strategy depends on various factors, including the number of energy sources (PV, Wind turbine, storage system), the complexity of the MPPT algorithms (P&O, FLC and the proposed hybrid P&O/FLC), and the sophistication of the control algorithms (Proposed algorithm). Also, Hybrid MPPT algorithms, which combine multiple MPPT techniques, require more computational resources compared to traditional single-source MPPT methods.

Real-time feasibility is crucial for ensuring the timely response of the power management controller to changes in environmental conditions (changes in weather conditions), and energy demand variations. For scalability, it refers to the ability of the proposed power management controller strategy to accommodate changes in system size (from 1 to 10 kW), and complexity and of course, as the number of energy sources increases, the computational demands on the controller may grow proportionally. Also, computational efficiency is essential for maximizing the utilization of available processing resources and minimizing energy consumption. Efficient proposed algorithms, and proposed optimization technique have reduced the computational workload and improve overall system performance.

## Simulation study

The bidirectional buck-boost converters play a crucial role in regulating and maintaining the DC bus voltage at the desired reference value of 24 V in a controlled and efficient manner. In this system, there are two converters—one dedicated to the batteries and the other to the supercapacitors. The converters are designed to adjust the output voltage to maintain a 24 V DC bus. The duty cycle of the converters is adjusted according to the difference between the actual and reference voltages in the control strategy. When the DC bus voltage is below 24 V, the converters boost it, and when it’s above 24 V, they buck it. This bidirectional operation allows for efficient control of the DC bus voltage.

**Figure 29:**
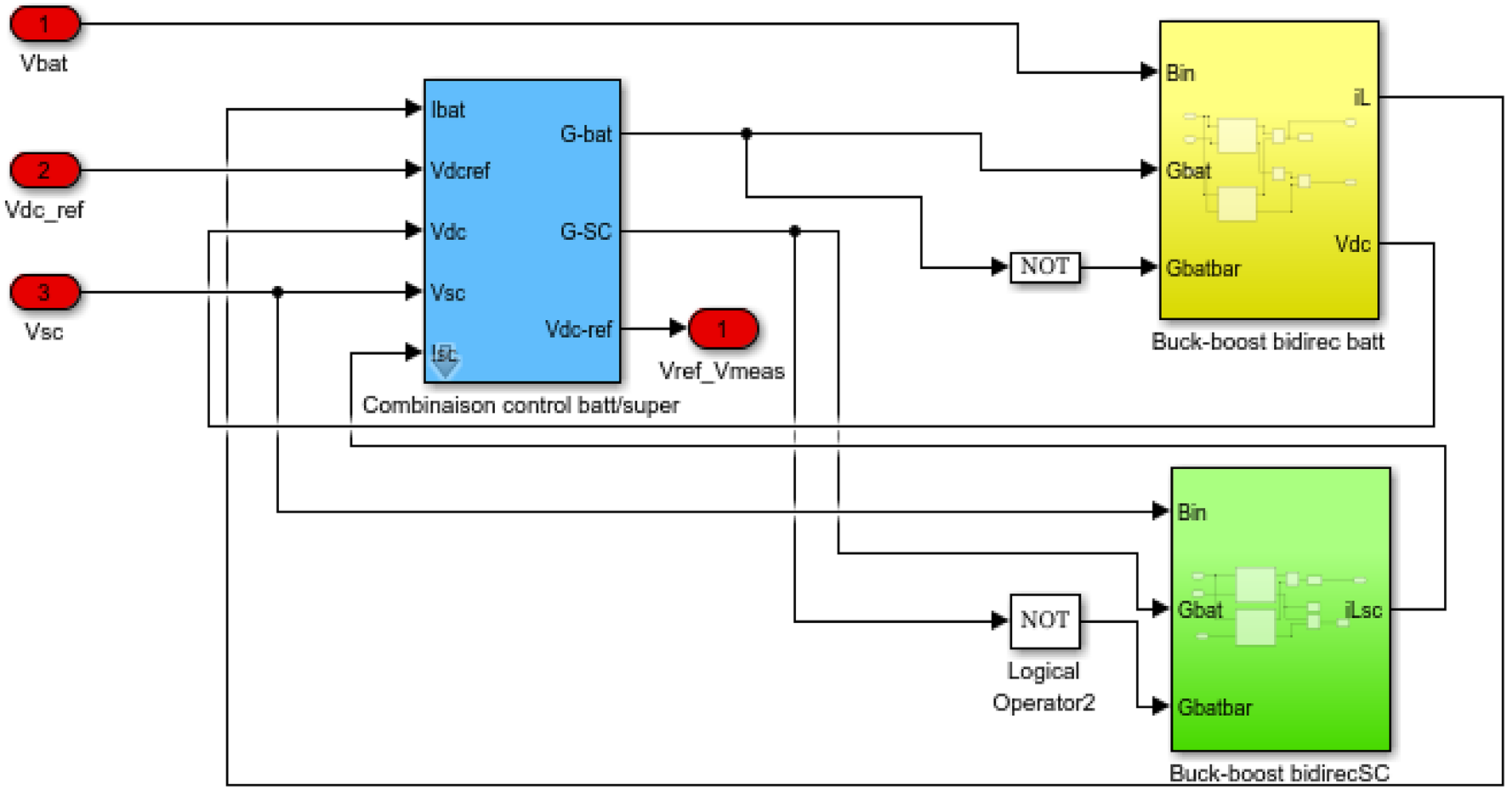
DC bus voltage calculation under Matlab/simulink.

**Figure 30:**
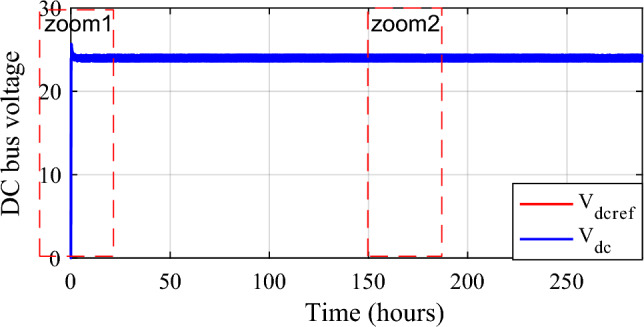
DC bus voltage and its reference.

**Figure 31:**
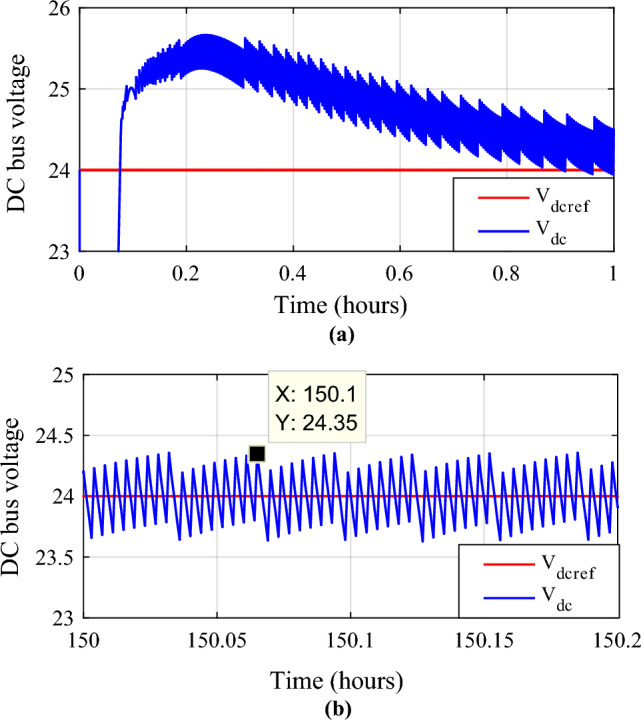
Zooms on DC bus voltage. (**a**) Zoom1. (**b**) Zoom2.

By having separate converters for batteries and supercapacitors, the system can efficiently manage the energy flow between these storage elements and the DC bus. This integration ensures optimal charging and discharging of both batteries and supercapacitors. The simulationresults have been presented and analyzed. Figures 29 and 30 show the DC bus voltage calculation under MATLAB/Simulink.The voltage on the DC bus closely matches the reference (Fig. 31a). It is controlled to the required voltage and keeps its reference (V_dcref_ = 24 V) with slight fluctuations with ΔV_dc_ = 0.36% < 1% (Fig. 31b). It is concluded that the voltage V_dcref_ matches the load demands while maintaining excellent control efficiency. This result demonstrates the efficiency of DC bus voltage control in ensuring optimal operation.

**Figure 32:**
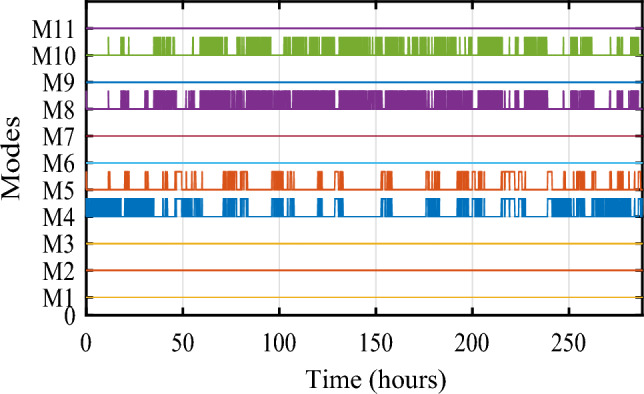
Eleven obtained modes.

**Figure 33:**
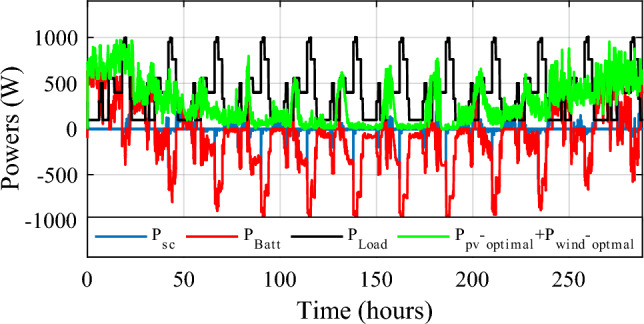
Different powers developed by the used power sources.

Figure 32 depicts the several modes that resulted and Fig. 33 illustrates simultaneous battery, supercapacitor, and PV power.

**Figure 34:**
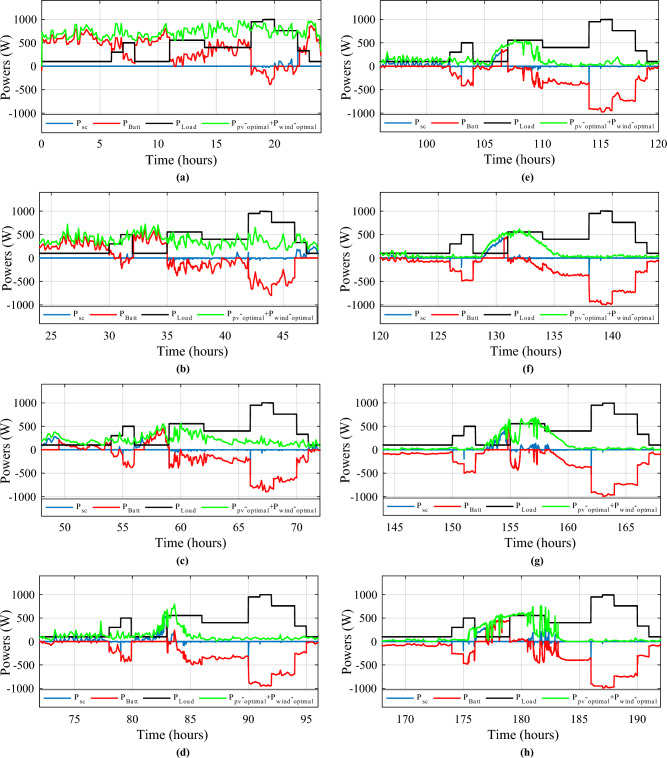

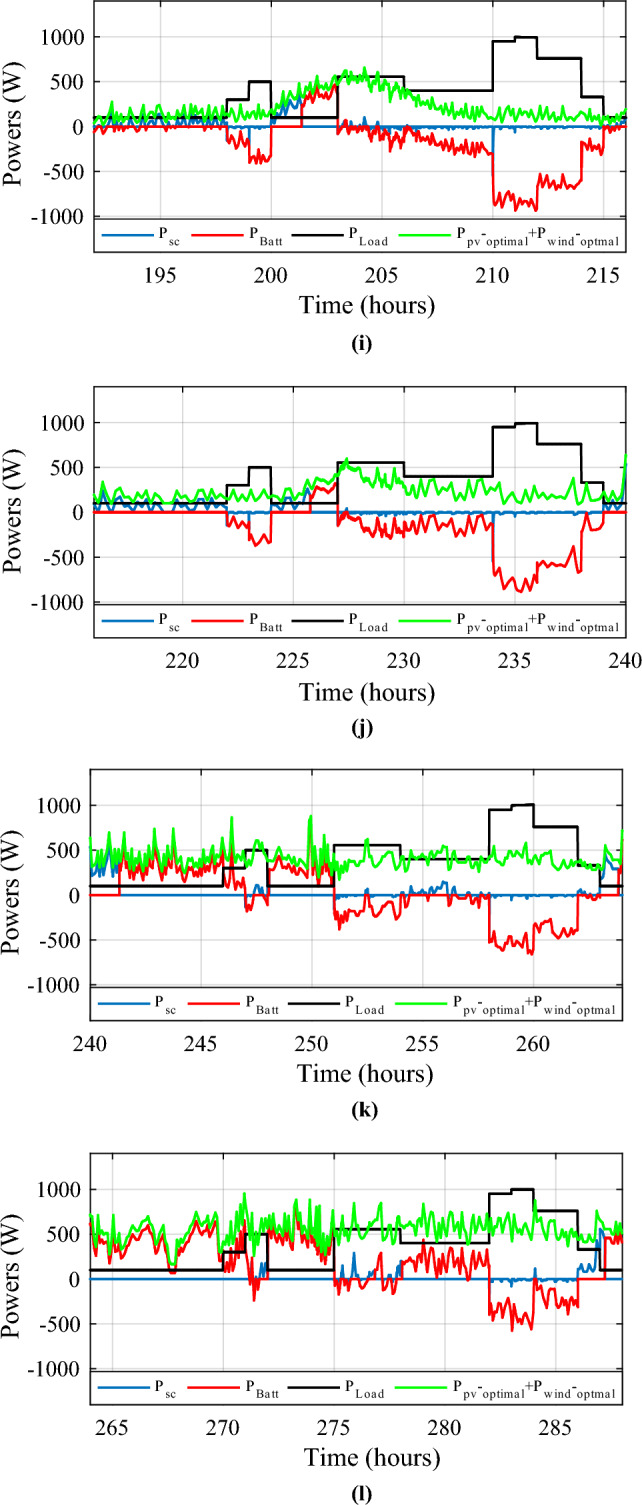
Developed powers per day. (**a**) Profile 1. (**b**) Profile 2. (**c**) Profile 3. (**d**) Profile 4. (**e**) Profile 5. (**f**) Profile 6. (**g**) Profile 7. (**h**) Profile 8. (**i**) Profile 9. (**j**) Profile 10. (**k**) Profile 11. (**l**) Profile 12

Figure 34 shows the daily power consumption throughout four different days. The PV and wind power profiles alter as the weather changes. It is observed that a negative curve for batteries and SCs indicates that they are recovering power, whereas a positive curve indicates that they are supplying the load.

**Figure 35:**
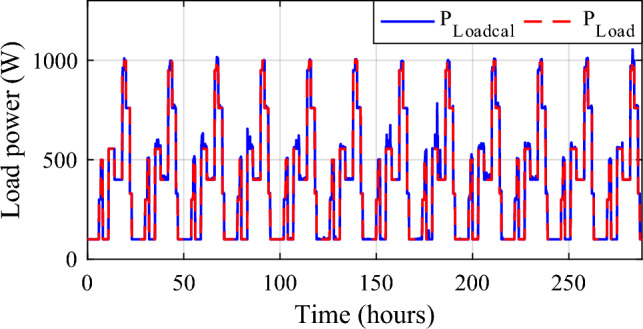
Calculated ${\text{P}}_{{{\text{Loadcalc}}}}$ and developed load power ${\text{P}}_{{{\text{Load}}}}$.

The battery receives substantial stress during the first two months, which are characterized by maximum average solar irradiances of 205.9 W/m^2^ and 465.7 W/m^2^ and significant wind speeds of 10.83 m/s and 7.76 m/s, respectively, and are supported by the SC during unexpected load shifts. M7, M8, M9, and M10 are the most often seen modes. Similar observations are taken for the third month, with maximum solar irradiation at 622.5 W/m^2^ and wind speed of about 8.26 m/s, but with fewer demands on the batteries, aided by the SC during sudden load changes.There is less demand on the batteries because the solar irradiance reaches 696.1 W/m^2^ on sunny days and up to 1000 W/m^2^ on cloudy days, until the wind speed drops from 8.87 m/s to 4.73 m/s. From the nine month (September) to the twelve one (December), it is noticed average solar irradiance varying respectively around the following average values 701 W/m^2^, 602.9 W/m^2^, 572.2 W/m^2^ and 789.2 W/m^2^ with an average wind speeds values 7.22 m/s, 7.93 m/s, 10.37 m/s and 13.15 m/s. These complementarities make less stress on the storage, where the batteries’ SOC has been kept between 73.5% and 90% while supercapacitor SOC was controlled between 42.39 and 90%. Figure 35 shows the reference load power as well as the total power generated by all power sources.

**Figure 36:**
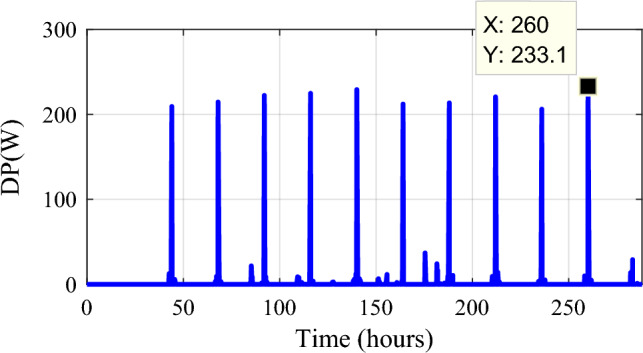
Gained power.

The calculated power sometimes surpasses the developed load power. The power excess has been computed (Fig. 36).

16 $$\Delta P_{Load} = P_{Loadcal} - P_{Load}$$

**Figure 37:**
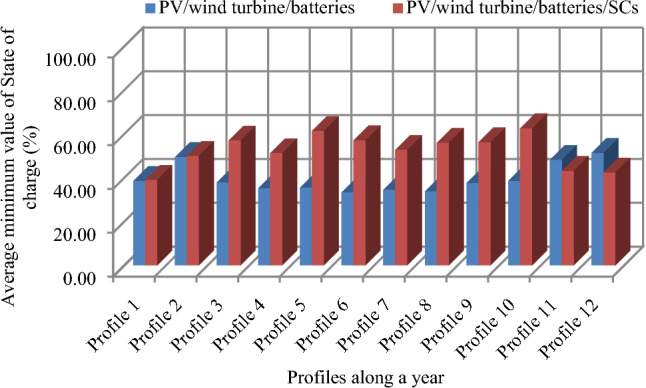
Evolution of the minimum state of charge under two cases along a year.

Despite the appropriate size and utilization of PMC, a small maximum power surplus is collected (233.1 W) during some profiles).To show the significance of system design choices and the impact on the battery’s SOC, which is crucial for the longevity and overall performance of the energy storage components, a comparison in terms of SOC evolution of the proposed system (PV/Wind turbine with hybrid storage) with a classical system with one storage (PV/wind turbine/batteries) has been made (Fig. 37).

**Figure 38:**
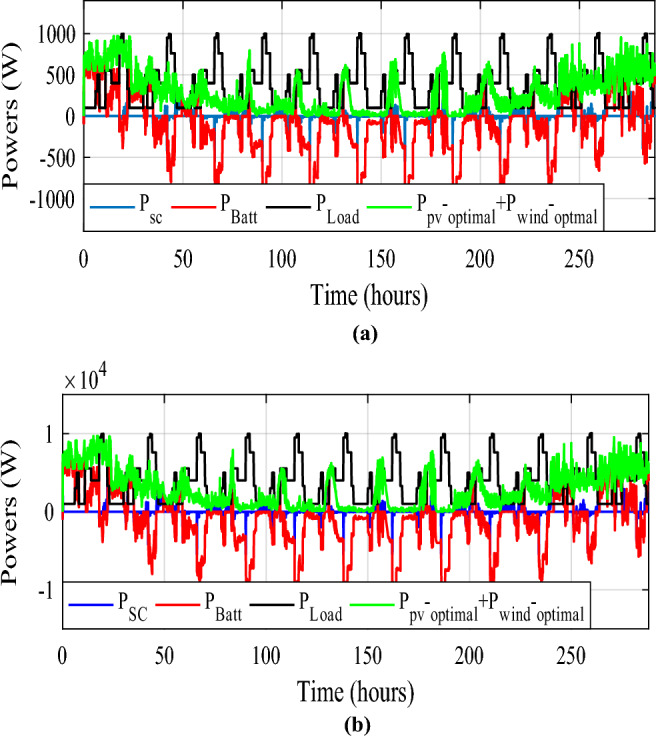
Scalability test on load power size. (**a**)Load power of 1 kW/day. (**b**) Load power of 10 kW/day.

**Figure 39:**
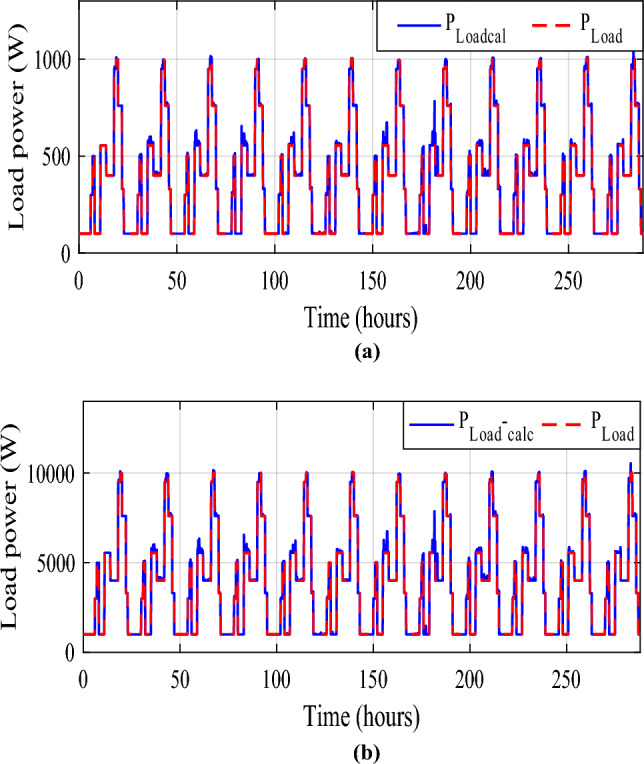
Scalability test on calculated load power size. (**a**)Load power of 1 kW/day. (**b**) Load power of 10 kW/day.

It is observed that the SOCmin in the traditional case (a PV/wind turbine system with batteries) varies between 33.15 and 51.16%, which is acceptable because it exceeds the algorithm’s 30% restriction. Battery stress decreases in the studied system, where the average SOC_min_ values vary between 38.87% and 62.42%. Inserting the SCs with the batteries is one of the better choices in areas with high solar radiation. Supercapacitors, with their high power density and rapid charge–discharge capabilities, can complement batteries by handling short-term power fluctuations effectively. We have tried to compare and evaluate how and whether the PMC strategy can scale effectively from small-scale installations to larger systems and we have re-size the system to supply a load of 10 kW, we have obtained the same results of course with greater powers, which confirm that the proposed system is scalable for all size of powers (Figs. 38 and 39).

## Real-time simulation

**Figure 40:**
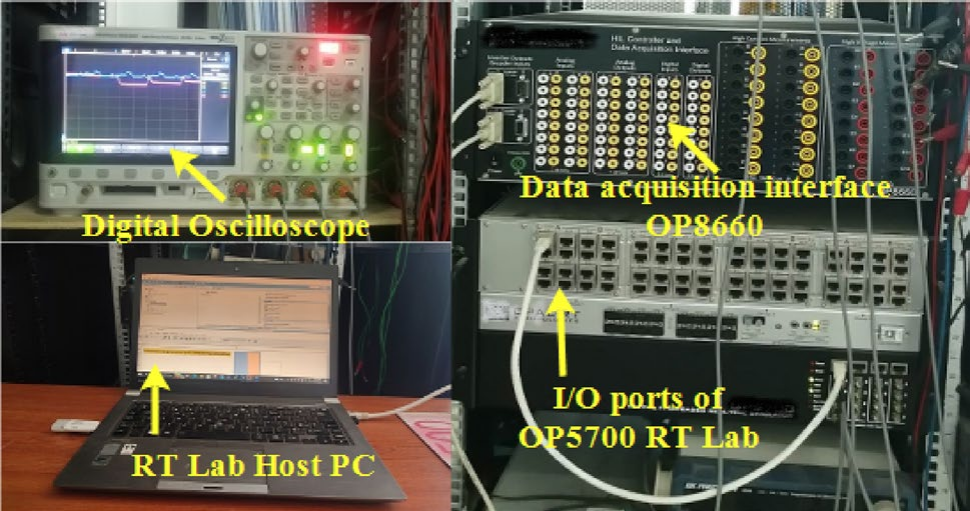
RT Lab real-time simulator work bench.

In order to endorse the numerical simulation results and to confirm them, a series of experimental tests were conducted on a real-time simulator (RT Lab) to assess the proposed coordinated power management strategy. The system settings remained constant, mirroring those used in the MATLAB/Simulink numerical simulation. As it may be noticed from Fig. 40, the real-time simulation bench consists of a host PC, a real-time digital simulator (OP5700), an HIL controller, an OP8660 data collection interface, and a digital oscilloscope.

**Figure 41:**
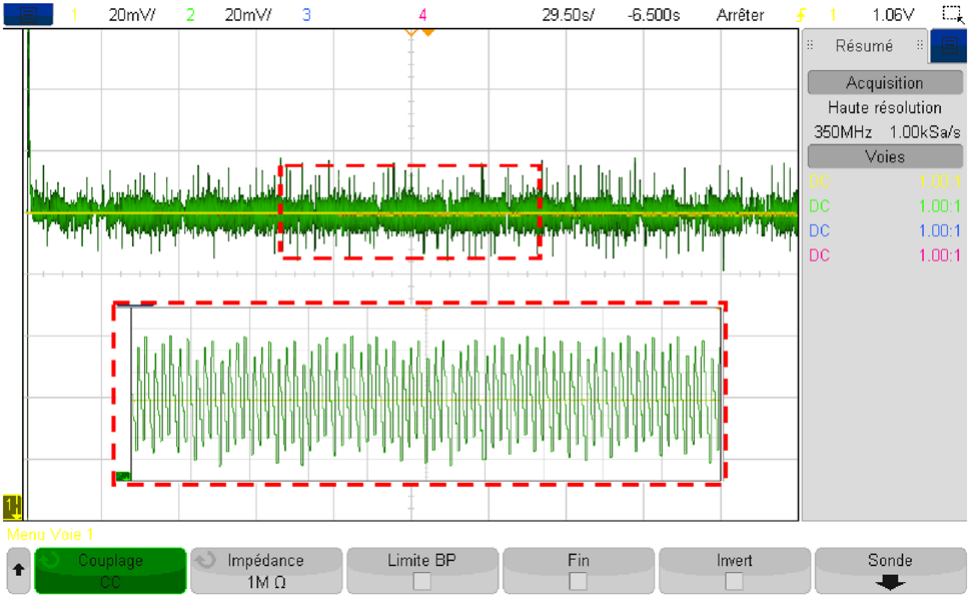
DC bus voltage in RTlab.

Figure 41 depicts the reference DC bus voltage, along with its reference and a zoomed-in view of the mentioned quantity. It is evident that V_DC_ tracks precisely its reference. Additionally, one can notice from the aforementioned figure that voltage ripples are contained within tolerable narrow band.

**Figure 42:**
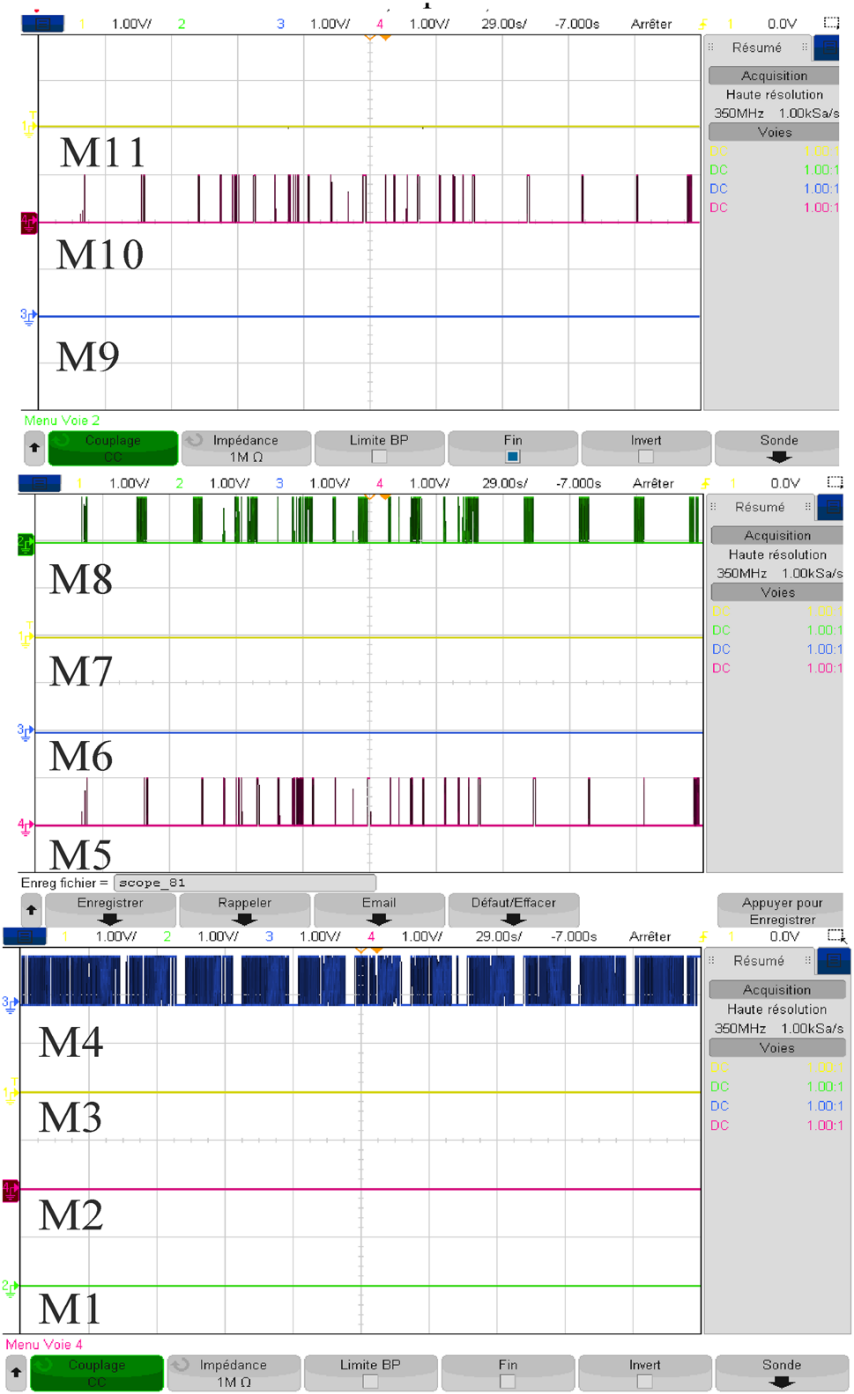
Different modes obtained in RTlab.

Figure 42 illustrates the different modes obtained when the proposed energy management strategy is executed using the RT LAB real-time simulation platform.

**Figure 43:**
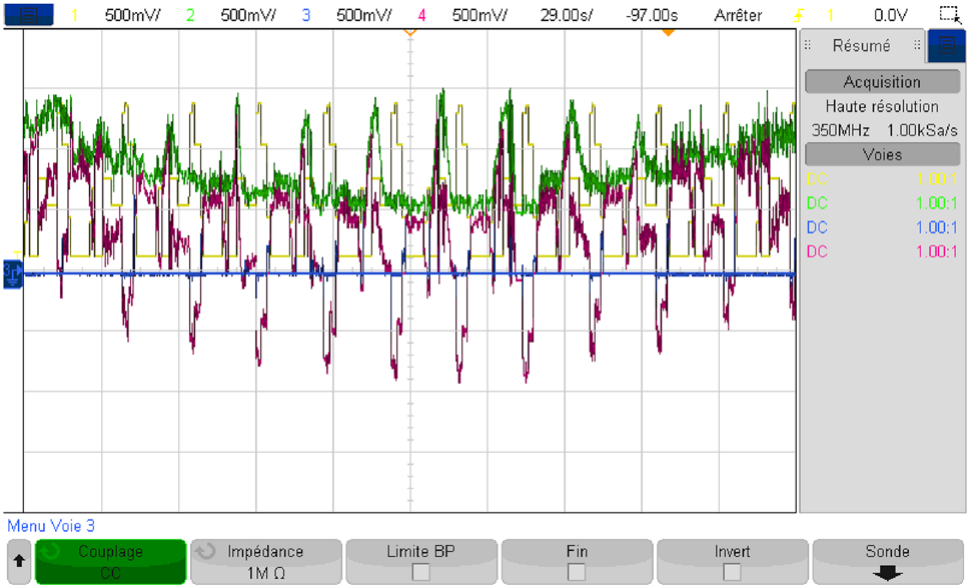
Power developed by each energy source in RTlab.

The power developed by each energy source is shown separately in Fig. 43.

**Figure 44:**
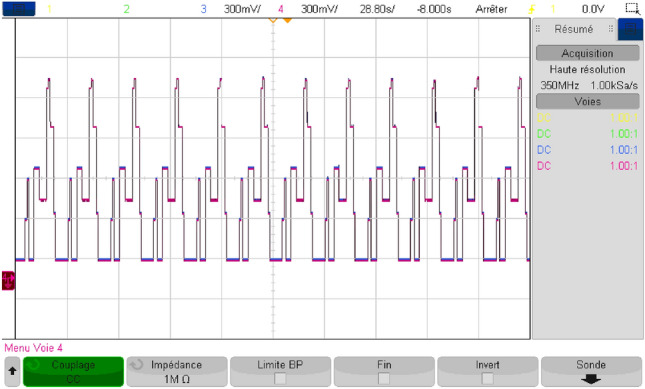
Load and developed load power in RTlab.

As shown in Fig. 44, the load power equals the developed load power.

**Figure 45:**
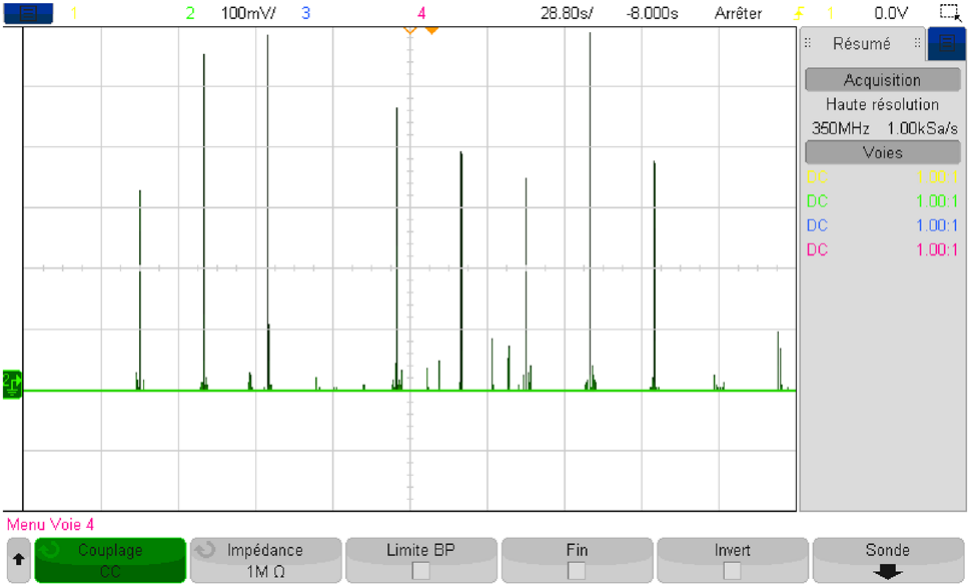
Gained power in RTlab.

This illustrates the effectiveness of the developed energy management strategy which makes power sources deliver exactly the required power without considerable losses. The obtained power gain was evaluated and represented in Fig. 45. This reflects the added value provided by the proposed coordinated energy management strategy and its ability to optimize the use of power sources.

The findings were validated through simulation using MATLAB/Simulink, and subsequently tested in real-time using the RT LAB simulation platform. This indicates that the utilized control method is effective, facilitating the proper flow of energy and ensuring optimal system operation.

## Economical study

**Figure 46:**
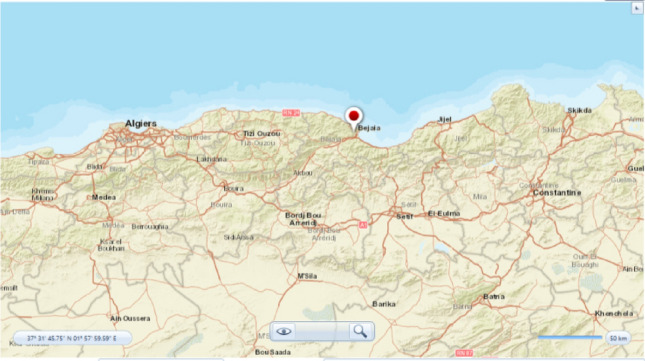
Bejaia geographical loation.

Economic factors, including capital costs, operational expenses and financing options are critical considerations in the practical implementation of hybrid multi-source systems. Economic feasibility assessments, including lifecycle cost analysis, return on investment calculations, and sensitivity analysis to varying input parameters, can help evaluate the economic viability of the system. This study has been made, where an economical consideration will be investigated examined using the Homer Pro program (Fig. 46).

**Figure 47:**
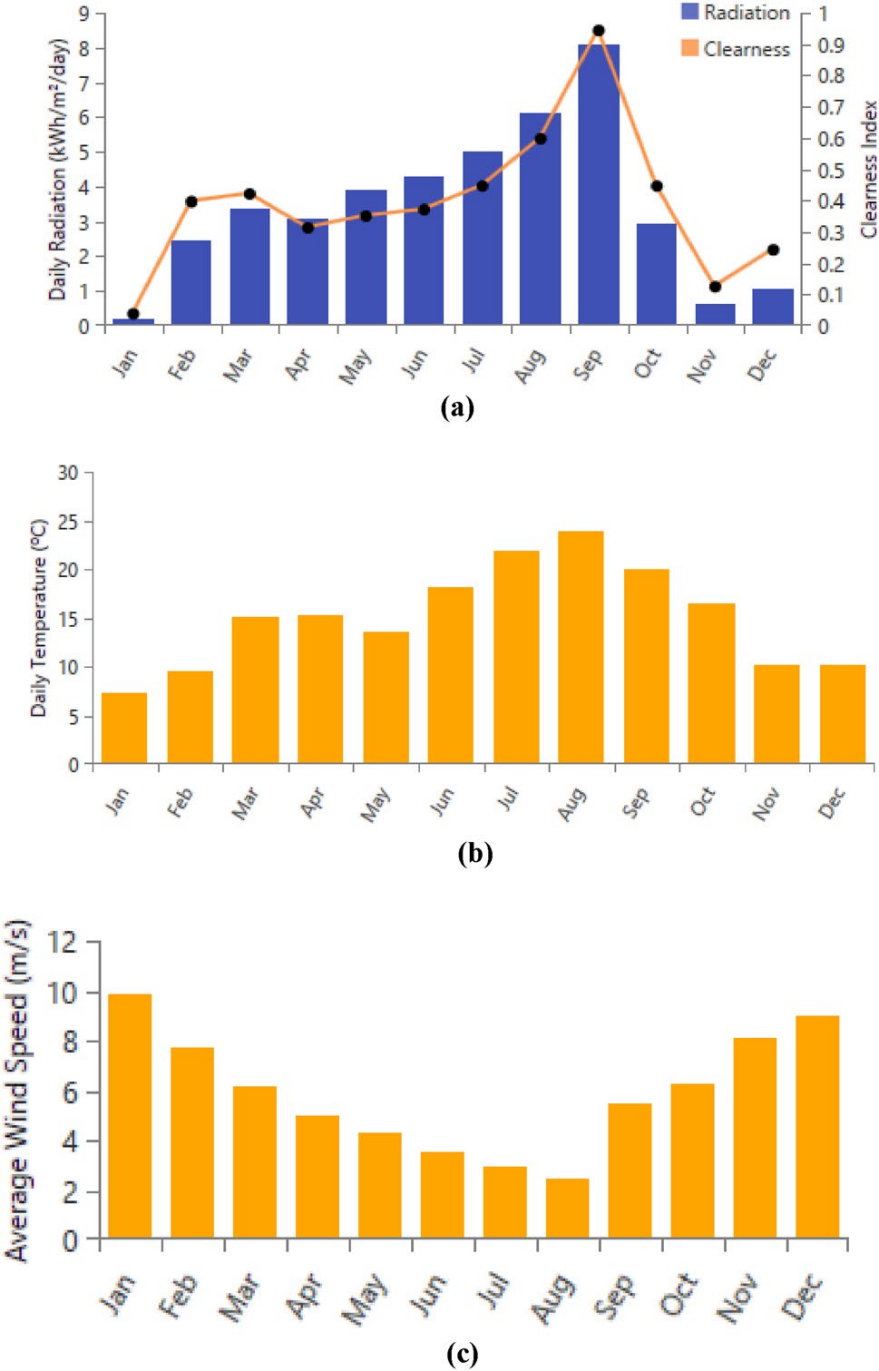
Weather conditions in Bejaia site. (**a**) Solar irradiance. (**b**) Ambient temperature. (**c**) Wind speeds.

**Figure 48:**
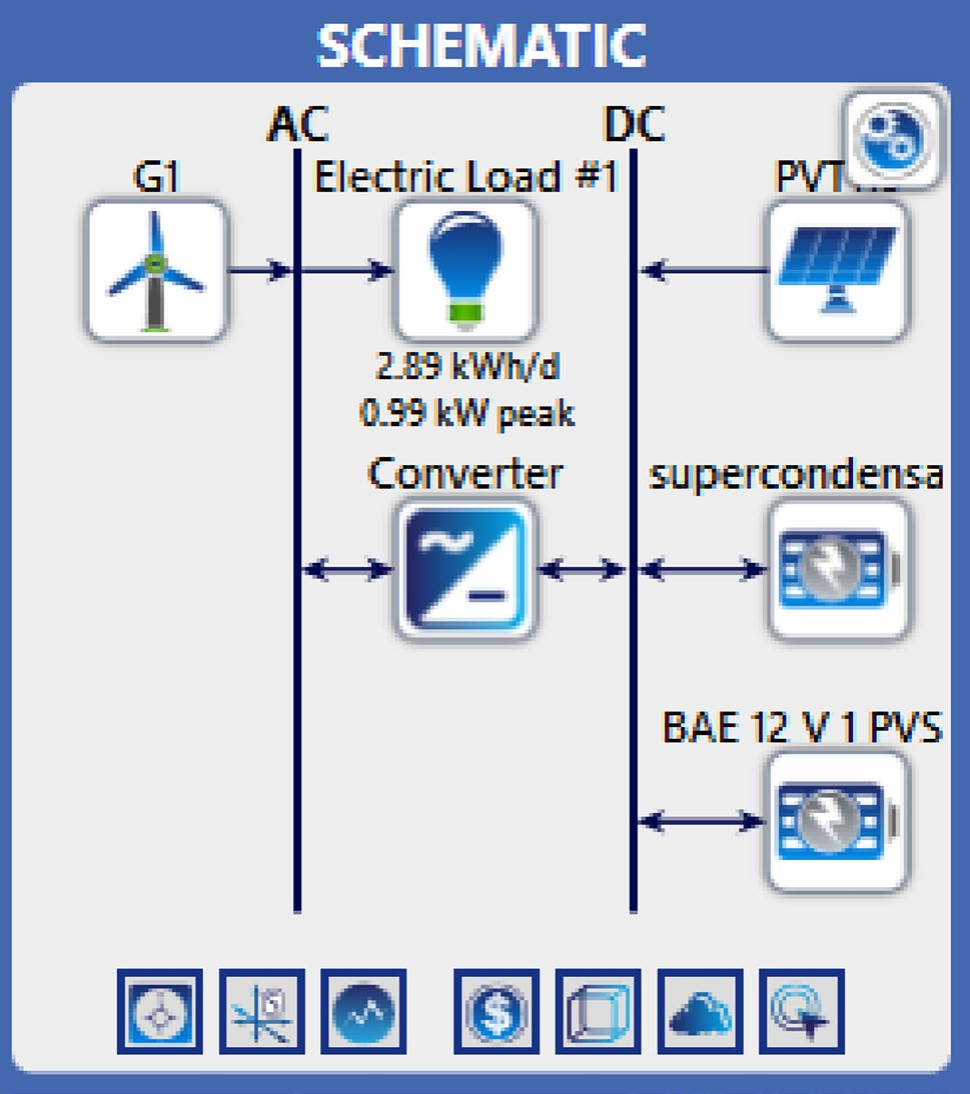
Configuration of the studied system.

**Figure 49:**
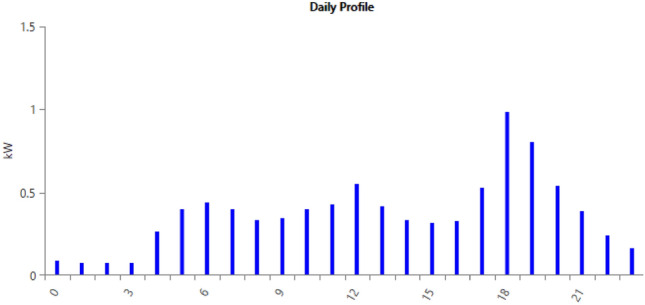
Load profile.

Figure 47 showcases the data for solar irradiance, wind speeds, and temperature, which were obtained utilizing Homer Pro software. Furthermore, Fig. 48 presents the design of the hybrid system in details. The load profile is given in Fig. 49 and the different components in Table 7.

**Table 7:** Inputs for the various components.

Component	Capital cost($)	O&M cost ($)	Replacement ($)	lifetime (years)
PV generator	1000.00	1.00	750.00	25
Wind turbine	3000.00	100.00	600	20
Batteries	167.00	8.00	167.00	10
SCs	60.00	0.00	45.00	20
Converter	400.00	9.30	300.00	20

The analysis took into consideration various economic factors such as system lifespan, initial costs, and maintenance costs. The cost of energy produced, which is represented by the cost of energy (COE), was also included in the assessment.

17 $${\text{COE}} = \frac{{{\text{C}}_{{{\text{tot}},{\text{ann}}}} }}{{{\text{E}}_{{{\text{tot}}}} }}$$

where: C_tot,ann_ is the total annual cost ($/year) of the hybrid energy system, E_tot_ the total annual electricity production (kWh/year).

Additionally, the net present cost (NPC) is HOMER’s main economic indicator, and all simulated systems are classified according to its value.

18 $${\text{NPC}} = \frac{{{\text{C}}_{{{\text{tot}},{\text{ann}}}} }}{{{\text{CRF}}\left( {{\text{i}},{\text{ t}}} \right)}}$$

where t is the project lifetime, i is the annual interest rate (%) and CFR is the capital recovery factor.

After simulation, the software suggested a more affordable architecture, with an NPC cost of $5914.81, a levelized COE of $439 and an operating cost of $144.29 (Table 8).

**Table 8:** Obtained simulation results.

Component	Capital ($)	Replacement ($)	O&M ($)	Salvage ($)	Total ($)
PV generator	702.32	0.00	8.98	0.00	711.30
Wind turbine	3000.00	187.08	1278.34	104.85	4360.57
storage	334.00	290.65	204.53	38.91	790.27
Converter	33.94	10.62	10.09	1.98	52.67
System	4070.26	488.35	1501.93	145.74	**5914.81**

**Figure 50:**
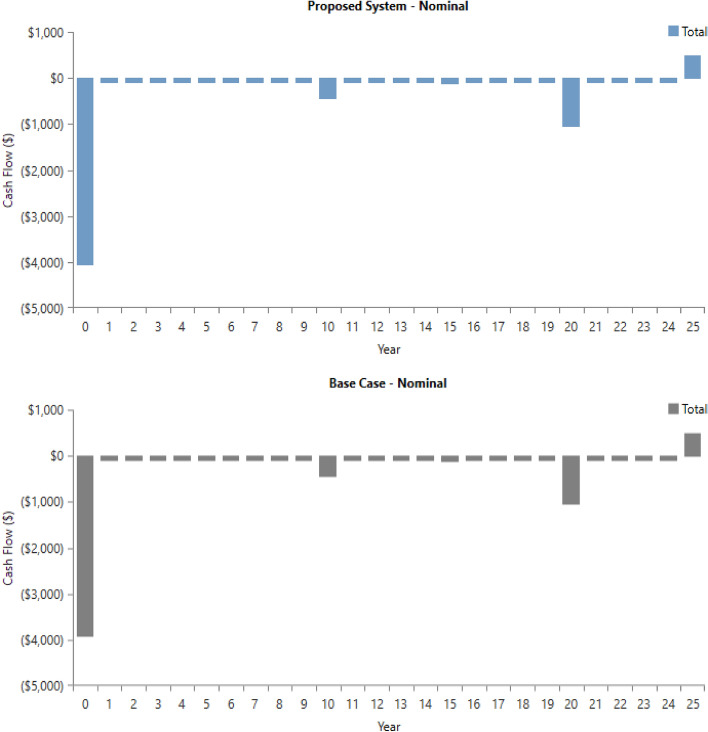
Cash flow of the proposed system with a base case in the software.

The software compare the cash flow of the proposed system with a base case in the software (Fig. 50).

The analysis was carried out in Bejaia, a location in North Algeria with readily available solar and wind data. The results indicate that the studied hybrid system is efficient with a residential electricity cost of $0.1 per kWh in Bejaia.

## Conclusion

The integration of renewable energy sources in islated locations using hybrid power optimization approaches and a multi-energy storage system with batteries and supercapacitors is discussed in this research paper. Our contribution on a Power Management Controller (PMC) and a multi-storage system integrated into a hybrid PV/Wind turbine system, optimized and validated through MATLAB/Simulink simulation and real time with RTlab, is a significant contribution in renewable energy systems.Thefindings show that the proposed PMC has successfully addressed weather conditions and geographic considerations, leading to high system performance throughout the year in the Mediterranean area. The reduction in stress on batteries, as compared to existing systems with only one storage (PV/Wind turbine/batteries), is a noteworthy advantage.

Some important practical implications can be on enhancing system efficiency, improving reliability, obtaining optimal power utilization, making battery management, adapting the studied system to variable environmental conditions, saving costs and of course reducing environmental impact. Indeed, a hybrid MPPT algorithm optimizes the power extraction from multiple sources like solar panels and wind turbines. This optimization leads to increased overall system efficiency by ensuring that each source operates at its maximum power point (MPP) under varying environmental conditions. Also, b**y** integrating multiple renewable energy sources (such as solar and wind), a hybrid system becomes more reliable. A well-designed power management strategy ensures that energy from different sources is efficiently utilized based on demand and availability. Excess energy from one source can be stored or redirected to other applications or storage systems.

As a further work, it is intended to use advanced intelligent techniques to enhance the performance of the proposed multi source renewable energy system. In addition to technical considerations, conducting an economic analysis would provide insights into the cost-effectiveness of the proposed configuration. This could include the initial setup costs, maintenance expenses, and the overall return on investment over the expected lifespan of the system.

## Data Availability

The datasets used and/or analysed during the current study available from the corresponding author on reasonable request.

## Abbreviations

AC: Alternate current
DC: Direct current
BMS: Battery management system
FCs: Fuel cells
FLC: Fuzzy logic controller
IPMC: Intelligent power management control
HESS: Hybrid energy storage system
HCS: Hill climbing search
HPV: Hybrid photovoltaic MPPT
HTb: Hybrid turbine MPPT
MPP: Maximum power point
MPPT: Maximum power point tracking
PEMFC: Proton exchange membrane fuel cell
P&O: Perturb & observe
PMSG: Permanent synchronous generator
PV: Photovoltaic
SC: Supercapacitor
SOC: State of charge
SOC_Batt_: Battery state of charge
SOC_SC_: Supercapacitor state of charge

## List of symbols

E_o_: Open circuit voltage (V)
e_pv_, e_Tb_: PV and turbine error
C: Capacity (F)
C_Batt_: Capacity battery (Ah)
Ce_pv_, Ce_Tb_: Photovoltaic and turbine variation of the error
C_p_: Power coefficient
C_pmax_: Optimal power coefficient
D: Duty cycle
D_pv_: PV duty cyle
D_Tb_: Turbine duty cyle
E_s_: Solar irradiance (W/m^2^)
I_Batt_: Battery current (A)
I_d_: Diode-current (A)
I_ss_, I_sq_: (d, q) stator currents (A)
I_dref_: Direct reference current (A)
I_DC_: Direct bus current (A)
I_L_: Inductance current (A)
I_pv_: PV current (A)
I_qref_: Quadrate reference current (A)
I_Rsh_: Shunt resistance current (A)
I_sc_: Supercapacitor current (A)
I_ph_: Photo-current (A)
K_dDpv_, K_dDTb_: PV and turbine proportionality constant
K_epv_, K_eTb_: Scaling factor of PV and wind error
KC_epv_, KC_eTb_: Scaling factor of PV and wind error variation
L: Inductance (H)
L_d_, L_q_: (d, q) inductances (H)
P: Pair pole number
P_Load_: Load power (W)
P_Load-calc_: Calculated load power (W)
P_MPP_: Maximum power point power (W)
P_pv_: Photovoltaic power (W)
P_pv-optimal_: Optimal photovoltaic power (W)
P_Tb_: Turbine power (W)
P_Tb-optimal_: Optimal turbine power (W)
R_Batt_: Internal battery resistance (Ω)
R_Tb_: Turbine radius (m)
R_st_: Stator windings resistance
T_a_: Ambient temperature (°C)
T_em_: Electromagnetic torque (N m)
T_eref_: Electromagnetic torque reference (N m)
U_sc_: Supercacitor voltage (V)
V_dc_: DC bus voltage (V)
V_dcref_: DC bus voltage reference (V)
V_Batt_: Battery voltage (V)
V_L_: Inductance voltage (V)
V_sd_, V_sq_: (d–q) Stator voltages (V)
V_wind_: Wind speeds (m/s)
Xe_pv_, Xe_Tb_: Normalized values of PV and turbine error
XCe_pv_, XCe_Tb_: Normalized values of PV and turbine error variation

## Greek letter

ΔP: Power demand variation (W)
ΔP_pv_: Photovoltaic power excess (W)
ΔP_Load_: Load power variation (W)
ΔP_wind_: Wind power excess (W)
ΔP_renew_: Renewable power excess (W)
ΔV_dc_: DC bus voltage variation (V)
ΔV_pv_: Photovoltaic voltage variation (V)
Φ_f_: Magnetic flux produced by the permanent magnet (Wb)
Φ_f_: Magnetic flux produced by the permanent magnet (Wb)
λ: Tip speed ratio
λ_opt_: Optimal tip speed ratio
ρ: Air density
Δω_Tb_: Wind turbine velocity variation (rad/s)
ω_Tb_: Wind turbine velocity (rad/s)
